# Evaluation of Anti-*Trypanosoma cruzi* Activity of Chemical Constituents from *Baccharis sphenophylla* Isolated Using High-Performance Countercurrent Chromatography

**DOI:** 10.3390/molecules29010212

**Published:** 2023-12-30

**Authors:** Matheus L. Silva, Felipe S. Sales, Erica V. C. Levatti, Guilherme M. Antar, Andre G. Tempone, João Henrique G. Lago, Gerold Jerz

**Affiliations:** 1 Center for Natural Sciences and Humanities, Federal University of ABC, Santo André 09210-580, Brazil; matheus.lopes@ufabc.edu.br (M.L.S.); felipe.sales@ufabc.edu.br (F.S.S.); 2 Laboratory of Pathophysiology, Butantan Institute, São Paulo 05508-040, Brazil; ericavclevatti@gmail.com (E.V.C.L.); andre.tempone@butantan.gov.br (A.G.T.); 3 Department of Agrarian and Biological Sciences, Federal University of Espírito Santo, São Mateus 29932-540, Brazil; guilherme.antar@ufes.br; 4 Institute of Food Chemistry, Technical University of Braunschweig, 38106 Braunschweig, Germany

**Keywords:** *Baccharis sphenophylla*, Asteraceae, diterpenes, flavonoids, *Trypanosoma cruzi*, high-performance countercurrent chromatography

## Abstract

Endemic in 21 countries, Chagas disease, also known as American Trypanosomiasis, is a neglected tropical disease (NTD) caused by the protozoan parasite *Trypanosoma cruzi*. The available drugs for the treatment of this disease, benznidazole and nifurtimox, are outdated and display severe side effects. Thus, the discovery of new drugs is crucial. Based on our continuous studies aiming towards the discovery of natural products with anti-*T. cruzi* potential, the MeOH extract from aerial parts of *Baccharis sphenophylla* Dusén ex. Malme (Asteraceae) displayed activity against this parasite and was subjected to high-performance countercurrent chromatography (HPCCC), to obtain one unreported *syn*-labdane diterpene — sphenophyllol (**1**) — as well as the known compounds gaudichaudol C (**2**), *ent*-kaurenoic acid (**3**), hispidulin (**4**), eupafolin (**5**), and one mixture of di-*O*-caffeoylquinic acids (**6**–**8**). Compounds **1**–**8** were characterized by analysis of nuclear magnetic resonance (NMR) and mass spectrometry (MS) data. When tested against trypomastigote forms, isolated labdane diterpenes **1** and **2** displayed potent activity, with EC_50_ values of 20.1 μM and 2.9 μM, respectively. The mixture of chlorogenic acids **6**–**8**, as well as the isolated flavones **4** and **5**, showed significant activity against the clinically relevant amastigotes, with EC_50_ values of 24.9, 12.8, and 2.7 μM, respectively. Nonetheless, tested compounds **1**–**8** displayed no cytotoxicity against mammalian cells (CC_50_ > 200 μM). These results demonstrate the application of HPCCC as an important tool to isolate bioactive compounds from natural sources, including the antitrypanosomal extract from *B. sphenophylla*, allowing for the development of novel strategic molecular prototypes against tropical neglected diseases.

## 1. Introduction

**Figure 1:**
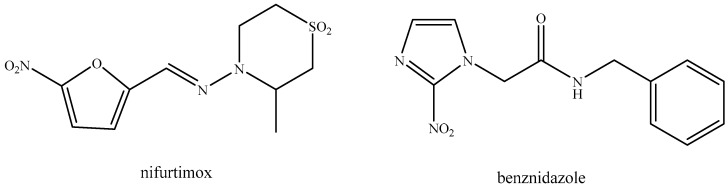
Structures of benznidazole and nifurtimox, the currently available drugs for the treatment of Chagas disease.

A concerning public health issue, Chagas disease, also known as American Trypanosomiasis, is a neglected tropical disease (NTD) caused by the protozoan parasite *Trypanosoma cruzi* [1]. Although primarily found in Latin America, where it is endemic in 21 countries, cases have also been reported to other regions due to migration and international travel [2,3,4]. According to the World Health Organization (WHO), there are currently around eight million people infected worldwide [5]. The available drugs for the treatments, benznidazole and nifurtimox (Figure 1), are outdated and display severe side effects [6,7]. 

According to the Drugs for Neglected Disease initiative (DND*i*), the effectiveness of benznidazole in treating the chronic phase of the disease and in preventing the development of cardiomyopathies or gastrointestinal pathologies is almost absent and still the subject of intense debate [8,9]. As such, the search for new alternative treatments, especially those based on natural products, is of utmost importance. While natural products can serve as valuable starting points for the development of new drugs [10], their prospective use still faces many limitations, such as the obtaining of reduced amounts of isolated compounds, the usage of high amounts of solvents, and time-consuming chromatographic procedures. In this regard, countercurrent chromatography (CCC)techniques that use solely liquid and immiscible solvent phase systems comprises a valuable method for the separation/isolation of natural products [11,12,13]. Additionally, CCC has been used for the separation and analysis of complex mixtures of compounds and offers several advantages over traditional chromatographic methods, such as high preparative resolution, high sample loading capacity, economic use and recycling of solvents, and the ability to separate compounds with similar properties [14,15]. As example, CCC has successfully been used by our research group for the isolation of anti-*T. cruzi* metabolites, including neolignans and derivatives from *Nectandra leucantha* [16,17].

Distributed throughout South America and especially in Brazil, the *Baccharis* genus is one of the most diverse groups of flowering plants belonging to the Asteraceae. Containing more than 500 recognized species, it is considered one of the largest genera within the family [18]. As previously reported by several phytochemical studies [19,20,21,22], the genus is known to afford a wide range of bioactive compounds, particularly terpenoids and flavonoids. Furthermore, some of these compounds have been found to display antitrypanosomal activity, such as those obtained from *B. sphenophylla* [23,24]. In the continuation of our studies, in the present work, the MeOH extract from the aerial parts of *B. sphenophylla* displayed activity against trypomastigote forms of *T. cruzi*. In order to isolate bioactive metabolites, this extract was initially chromatographed over Sephadex LH-20, and the obtained fractions were purified by high performance countercurrent chromatography (HPCCC). Using this approach, one new labdane diterpene, named sphenophyllol (**1**), as well as the known compounds gaudichaudol C (**2**), *ent*-kaurenoic acid (**3**), hispidulin (**4**), eupafolin (**5**), and one mixture of di-*O*-caffeoylquinic acids (**6**–**8**) were isolated. Furthermore, the cytotoxicity for NCTC cells and the effects against trypomastigote and amastigotes forms of the parasite *T. cruzi* of compounds **1**–**8** were evaluated in vitro. 

## 2. Results and Discussion

**Figure 2:**
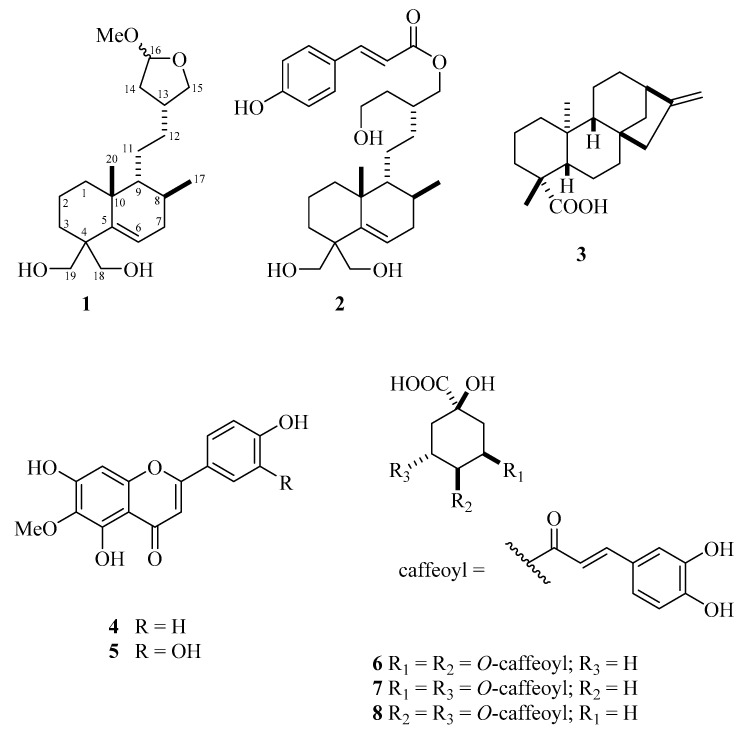
Structures of compounds **1**–**8** isolated from *B. sphenophylla*.

The screening against trypomastigotes of *T. cruzi* indicated activity for the MeOH extract from the aerial parts of *B. sphenophylla* (100% parasite death at 300 μg/mL). Based on this result, the extract was subjected to a chromatographic fractionation over Sephadex LH-20 and HPCCC, to afford compounds **1**–**8** (Figure 2).

NMR and ESI-MS spectral data analysis followed the comparison with those reported in the literature, allowing for the identification of gaudichaudol C (**2**) [25], *ent*-kaurenoic acid (**3**) [26], hispidulin (**4**) [26], eupafolin (**5**) [27,28] and one mixture of di-*O*-caffeoylquinic acids (**6**–**8**) [29,30]. Compounds **3**–**8** were previously isolated from *B. sphenophylla* [23,24,31], with this research being the first report of compound **2** in the studied plant.

Compound **1** was isolated as a white amorphous solid. The IR spectrum showed absorption bands at ν_max_ 3412 and 1660 cm^−1^ of the hydroxyl group and double bonds, respectively. The ESI-HRMS spectrum showed the [M + Na]^+^ ion peak at *m/z* 375.2515 (calc. for C_11_H_36_O_4_Na, 375.2511), indicating, for compound **1**, the molecular formula C_21_H_36_O_4_, with four unsaturation. ^1^H NMR data of compound **1** indicated characteristic signals for a labdane-type diterpene, similar to those reported in gaudichaudol A [25], such as a triplet at δ 5.69 (*J* = 3.6 Hz, H-6) and two superimposed double-doublets at δ 4.96 (*J* = 5.5 and 2.7 Hz, H-16). The signals at δ 4.14 (d, *J* = 11.8 Hz), 3.80 (d, *J* = 11.8 Hz), 3.96 (d, *J* = 10.7 Hz), and 3.56 (d, *J* = 10.7 Hz) correspond to the methylene hydrogens H-18α, H-18β, H-19α, and H-19β, respectively, as also observed for the chemically related diterpene gaudichaudol C (**3**) [25]. This spectrum also showed two double-doublets at δ 3.99 (*J* = 15.7 and 8.0 Hz) and 3.40 (*J* = 16.2 and 8.1 Hz), corresponding to hydrogens H-15α and H-15β and two singlets at δ 3.32 and 3.29, suggesting the presence of epimerized methoxy groups at C-16. Additional peaks at δ 0.83 (d, *J* = 6.4 Hz, 3H) and δ 0.76 (s, 3H) were assigned to H-17 and H-20, respectively. 

^13^C NMR and DEPT spectra of compound **1** showed two peaks corresponding to *sp^2^* carbons at δ 148.1 (C) and 128.7 (CH), which were attributed to C-5 and C-6, respectively. Two duplicated signals at δ 106.6/107.0 (C-16) and δ 73.2/73.6 (C-15) were also observed, suggesting the presence of an epimeric cyclic acetal containing a methoxy group at C-16, observed at δ 54.7/55.2, similarly to the clerodanes isolated from *B. gaudichaudiana* [32] and *B. articulata* [33]. Two oxymethylene carbon peaks, at δ 65.7 and δ 64.5, were assigned to C-19 and C-18, respectively, as also observed in gaudichaudol C [25]. Additionally, the signals at δ 19.3 and 16.2 were attributed to the methyl groups C-17 and C-20, respectively. Such connectivity was determined based on the correlations observed in the HMBC spectrum between the signals of H-18/H-19 and C-5, H-15 and C-12/C-13/C-14, and MeO and C-16. Therefore, these data confirmed the positioning of the methoxytetrahydrofuran group at C-12, the double bond at C-5, and two hydroxyl groups at C-18 and C-19. Analysis of the HSQC and HMBC spectra enabled the complete assignment of all hydrogens and carbons of compound **1**. Finally, the relative stereochemistry of **1** was assigned on the basis of analysis of the NOESY spectrum, which showed strong correlations between H-9 and H-20 and H-17, indicating that both methyl groups are axial orientated, relative to the decalin unit, in order to configure a *syn*-type labdane [34,35]. Finally, considering that compounds **1** and **2** are probably formed by one unique biosynthetic precursor, the same configuration was suggested for C-13 to both diterpenes. All of these data confirmed the structure of compound **1** as 15,16-epoxy-18,19-dihydroxy-16-methoxy-*syn*-labd-5-ene, named sphenophyllol. However, given that MeOH was used in the extraction, in chromatographic procedures, and to record NMR spectra, compound **1** could be considered an artefact and the true natural product is its hemiacetal analogue. In order to confirm this hypothesis, the hexane extract from aerial parts of *B. sphenophylla* was dissolved in EtOAc and directly analyzed by ESI-HRMS. As a result, a peak at *m/z* 375.2516, corresponding to an [M + Na]^+^ ion, was detected. Therefore, it is possible to conclude that **1** is an undescribed natural product. 

The mammalian cytotoxicity of compounds **1**–**8** was evaluated in the murine fibroblasts of NCTC cells. As shown in Table 1, all tested compounds, as well as the mixture (**6**–**8**), displayed no toxicity, to the highest tested concentration of 200 μM. Sequentially, the respective EC_50_ values against both trypomastigote (extracellular) and amastigote (intracellular) forms of *T. cruzi* of compounds **1**–**8** were determined, and the obtained results are presented in Table 1. These data indicated that chemically related diterpenes **1** and **2** displayed activities against trypomastigotes with EC_50_ values of 20.1 and 2.9 μM, respectively. Considering the chemically related structures of both of these diterpenes, it is possible to suggest that the presence of caffeoyl moiety in the structure of compound **2** plays an important role in its bioactivity, considering that caffeic acid was reported to display potential against trypomastigotes of *T. cruzi* [29]. The selectivity index, given by the ratio between the mammalian cytotoxicity and the antiparasitic activity, showed that compound **2** presented a value of SI > 68.7. Additionally, it exhibited six-fold higher potency than the standard drug benznidazole, which demonstrated an EC_50_ of 18.7 μM against the trypomastigotes and a SI > 10.7. Despite this effect, both compounds **1** and **2** were inactive when tested against the amastigotes. This could be a result of denaturation of the compound after penetration into the host cell or a lack of penetration in the macrophages. Compounds containing a hydroxycinnamic group analogue to that found in compound **2** are known to exhibit anti-*T. cruzi* activity, such as the phenylpropanoids isolated from *B. sphenophylla* [23] and *B. ligustrina* [36]. 

Compound **3** displayed moderate activity against trypomastigote forms, with an EC_50_ value of 10.6 μM, but it was inactive against the intracellular amastigotes. One of the most promising results is the data presented for flavones **4** and **5**, which displayed the highest potency against the intracellular amastigotes, with EC_50_ values of 12.8 and 2.7 μM, respectively. Interestingly, the higher effect was observed in compound **5**, which displayed a catechol moiety in ring B instead of a phenolic unit, as observed in the less active compound **4**. The selectivity index values for amastigotes were >12.6 and >74.1, respectively, confirming the promising antitrypanosomal potential. Both compounds showed no activity against the trypomastigotes. These differences are normal in drug discovery studies for *T*. *cruzi* and can be explained by the differential metabolism of trypomastigotes (extracellular forms) when compared to the intracellular forms, which results in the differential susceptibility of the parasites to chemical compounds. The EC_50_ value of compound **5** was similar to the standard drug benznidazole (EC_50_ = 5.5 μM) against the amastigotes. The in vitro efficacy toward the intracellular amastigotes is the main goal of drug discovery studies for *T. cruzi* and a selectivity index > 10 is considered to be one of the major criteria for the selection of new hit compounds by the DND*i* [37]. 

The mixture characterized as compounds **6**–**8** displayed no activity against trypomastigote forms of *T. cruzi* [29] but exhibited moderate potential against amastigote forms, with an EC_50_ value of 24.9 μM, resulting in a SI > 8.0. 

Therefore, it is possible to conclude that flavonoids **4** and **5** fulfill the desired criteria for the discovery of new hit compounds for Chagas disease, as suggested by the Drugs for Neglected Diseases initiative (DND*i*). Additionally, the DND*i* also suggests that candidate compounds present activity against the intracellular amastigotes of the parasite with a selectivity index of at least 10, which indicates a ten-fold higher effectiveness against the parasite than toxicity for mammalian cells [38]. 

## 3. Materials and Methods

### 3.1. General Experimental Procedures

IR spectra were obtained in a Perkin-Elmer infrared spectrometer model 1750. NMR spectra data were recorded on a Bruker FT 300 (Bruker Biospin, Ettlingen, Germany) or on a Varian Inova 500 (Varian, Palo Alto, CA, USA) spectrometer, operating at 300/500 MHz (^1^H) and 75/125 Hz (^13^C), respectively, using CDCl_3_, DMSO-d_6_, or CD_3_OD (Deutero GmbH, Kastellaun, Germany) as solvents and TMS (Sigma-Aldrich, Deisenhofen, Germany) as an internal standard. Chemical shifts (δ) are given in ppm, with coupling constants (*J*) in Hz. HRMS and LRMS spectra were performed, respectively, on a TIMS-Q-TOF Maxis 3G spectrometer and on an Esquire 3000 Plus spectrometer (Bruker Daltonics, Billerica, Massachusetts, USA), operating with electrospray ionization (ESI) in positive or negative modes. Optical rotation was measured in a digital polarimeter JASCO DIP-370 (Na filter, λ = 588 nm). Column chromatography (CC) was performed with Sephadex LH-20 (Sigma-Aldrich), while thin layer chromatography (TLC) separations were carried out on silica gel 60 PF_254_ (Merck, Darmstadt, Germany). Analytical grade (Labsynth Ltd., São Paulo, Brazil) solvents were used in extraction and chromatographic procedures.

### 3.2. Solvent System Selection and Determinaiton of Distribution Coefficient (K_D_) 

For the solvent system selection, the G.U.E.S.S. approach, with respective HEMWat systems, was chosen [39]. To perform this initial test, 50 mg of the target fraction was dissolved in 10 mL of CH_2_Cl_2_. Aliquots of 1 mL were dispensed using a volumetric pipette into 10 mL marked vials and were left to evaporate at room temperature. Mixtures of hexane/EtOAc/MeOH/H_2_O (HEMWat) were prepared with different proportions and, afterwards, 1 mL from each upper and lower phase of the prepared mixtures were added into the marked vials. Afterwards, the marked vials were shaken, homogenized, and observed for the settling time of the phases, according to estimate values described by the literature [39]. Using volumetric capillaries, aliquots of each upper and lower phase were spotted, eluted, and revealed on TLC. The solvent system was chosen based on the distribution of mixture components among both phases. The distribution coefficient (K_D_) was calculated according to the retention equation for CCC [40], as shown in Equation (1):

V_R_ = V_M_ + K_D_V_S_ (1)

where V_R_ is the retention volume of the solute, V_M_ is the volume of the retained mobile phase, and V_S_ is the volume of the retained stationary phase. Rearranging Equation (1), we can determine K_D_, as per Equation (2):

K_D_ = (VR − VM)/Vs (2)

Stationary phase retention (S_F_) as an indicator of column resolution [40] was determined by achieving hydrodynamic equilibrium and by following Equation (3):

S_F_ = (V_C_ − V_M_)/V_C_ × 100% (3)

where V_C_ stands for the volume of the column. 

### 3.3. High-Performance Countercurrent Chromatography, Spiral Coil Apparatus, and Peripheral Devices 

Separation procedures were carried out using a multilayer coil planet J-type high-performance countercurrent chromatography system (HPCCC centrifuge, model: Spectrum, Dynamic Extractions, Gwent, UK). The centrifuge was equipped with two self-balanced coil bobbins and had a β-value range of 0.52–0.86 (calculated using the equation β = coil radius r/revolution radius R). Two semi-preparative tube column systems, made of polytetrafluoroethylene (PTFE) with a bore size of 1.6 mm, were connected in series to create a coil column with a volume (V_C_) of 125 mL. The separation process was conducted in the head-to-tail (reversed-phase) mode using the evaluated solvent system, where the denser aqueous phase served as the mobile phase and the upper more organic layer as the stationary phase. The periphery of the coils housed the head-end of the coil configuration. The separation was performed at the maximum velocity of 1600 rpm (g-force level 243) on the HPCCC machine. A liquid cooling thermostat (RC6, Lauda Dr. Wobser GmbH & Co. KG, Lauda-Königshofen, Germany) was used to maintain a constant temperature of 30 °C within the HPCCC. Prior to use, the different solvents were equilibrated in a separatory funnel, and the two-phase layers were separated. Freshly prepared solvent layers were then pumped using a preparative LC pump (solvent delivery system, K-501, Knauer Wissenschaftliche Geräte GmbH, Berlin, Germany). The flow rate for the mobile phase during the HPCCC experiment was set to 5.0 mL/min. The hydrodynamic equilibrium was established and the stationary phase retention (S_F_) was measured for each run (not accounting for the dead volume of connecting periphery tubing). The samples were dissolved in 2.5 mL aliquots of the phase layers, filtered through a Chromafil Xtra GF-100/25 fiberglass membrane disc filter (1 μm pore size, 25 mm i.d., Macherey-Nagel, Düren, Germany), and injected into the separation column through a 5.0 mL sample loop, using a manual low-pressure sample injection valve (Rheodyne, Cotati, CA, USA). Detection was performed at 210 nm using a UV-detector K-2501 (Knauer Wissenschaftliche Geräte GmbH Berlin, Germany). The fraction collector SuperFrac type B racks (Pharmacia, Uppsala, Sweden) collected fractions every minute during the elution process. 

### 3.4. Plant Material and Extraction

Aerial parts of *B. sphenophylla* were collected at Campos do Jordão, São Paulo State, in May 2015. The plant material was identified by MSc. Guilherme M. Antar and received registration code SISGEN A4123E4. A voucher specimen has been deposited at the Herbarium (SPF) of the Biosciences Institute from Universidade de São Paulo (IB-USP), under the number SPF 2206669. Dried and milled plant material (220 g) was sequentially extracted with hexane (10 × 500 mL) and MeOH (15 × 500 mL). After solvent evaporation under reduced pressure, we obtained 15 g and 34 g of hexane and MeOH extracts, respectively.

### 3.5. Fractionation of MeOH Extract of B. sphenophyllla 

Part of the MeOH extract (5 g) was chromatographed over Sephadex LH-20 (h = 130 cm; Ø = 5 cm), eluted with MeOH, to obtain 400 fractions (4 mL each) which were pooled together in sixteen groups (A–P) after TLC analysis, eluted with mixtures of CHCl_3_/EtOAc 7:3, CH_2_Cl_2_/MeOH/H_2_O 75:25:1 or EtOAc/MeOH 1:1. Plates were exposed to UV light at 254 nm and 365 nm and subsequently sprayed with anisaldehyde/H_2_SO_4_/AcOH, followed by heating to 110 °C on a hot plate.

Groups D, E, and M, obtained from the fractionation over Sephadex LH-20, were individually injected into the 125 mL coil HPCCC system over a low pressure UpChurch injection valve (Cotati, USA), at a velocity of 1600 rpm and flow rate of 4 mL/min. 

Part of group D (500 mg) was injected into the HPCCC system using a biphasic solvent system mixture of hexane/EtOAc/MeOH/H_2_O 4:6:5:5 (*v*/*v*/*v*/*v*). The hydrodynamic equilibrium led to a stationary phase retention (S_F_) of 66%, and the residual stationary phase volume (V_S_) was calculated to be 83 mL. The extrusion mode started at 1 h 10 min from the start of the run. This process resulted in 110 fractions (4 mL) which were combined into 14 subgroups (D-1–D-14) after TLC analysis. Fractions from subgroup D-9 (19 mg), eluted at a retention volume (V_R_) of 230 mL, configuring a distribution coefficient (K_D_) of approximately 2.2, were composed by **1**. 

Part of group E (1500 mg) was injected into the HPCCC system using a biphasic solvent system mixture of hexane/EtOAc/MeOH/H_2_O 7:3:7:3 (*v*/*v*/*v*/*v*). The hydrodynamic equilibrium led to a stationary phase retention (S_F_) of 85.6%, and the residual stationary phase volume (V_S_) was calculated to be 107 mL in the HPCCC system. The extrusion mode started at 1 h 10 min from the start of the run. This process resulted in 120 fractions (4 mL), which were combined into 14 subgroups (E-1–E-14) after TLC analysis. Subgroup E-2 (400 mg), obtained from the aforementioned run, was injected into the HPCCC using a biphasic solvent system mixture of hexane/EtOAc/MeOH/H_2_O 4:6:4:6 (*v*/*v*/*v*/*v*). The hydrodynamic equilibrium led to a stationary phase retention (S_F_) of 64%, and the residual stationary phase volume (V_S_) was calculated to be 80 mL. The extrusion mode started at 1 h 10 min from the start of the run. This process resulted in 100 fractions (4 mL) that were combined into 14 subgroups (E-2-1–E-2-14). Fractions from subgroup E-2-6 (43 mg), eluted at a retention volume (V_R_) of 181 mL and configuring a distribution coefficient (K_D_) of approximately 1.70, were composed by **2**. Subgroup E-12 (33 mg), eluted at a retention volume (V_R_) of 392 mL in extrusion mode, was composed by **3**. 

Part of group M (250 mg) was injected into the HPCCC using a biphasic solvent system mixture of hexane/EtOAc/MeOH/H_2_O 3:7:5:5 (*v*/*v*/*v*/*v*). The hydrodynamic equilibrium led to a stationary phase retention (S_F_) of 88%, and the residual stationary phase volume (V_S_) was calculated to be 110 mL. Extrusion mode started at the tube 1 h and 8 min from the start of the run. This process afforded 110 fractions (4 mL) that were combined into 12 subgroups (M-1-M-12) after TLC analysis. Subgroups M-2 (80 mg), M-4 (46 mg), and M-6 (1.4 mg), eluted at retention volumes (V_R_) of 56, 119, and 155 mL, configuring distribution coefficients (K_D_) of 0.37, 0.95, and 1.27, were shown to be composed by **6**–**8**, **5,** and **4,** respectively. 

*Sphenophyllol* (**1**). [α]_D_^25^ –2.62 (*c* 0.125, MeOH). White amorphous solid. ESI-HRMS (positive mode) *m/z* 375.2515 [M + Na]^+^ (calcd. for C_21_H_36_O_4_Na, 375.2511). ^1^H NMR (CD_3_OD, 300 MHz): δ 5.69 (t, *J =* 7.3 Hz, H-6), 4.92 (dd, *J* = 5.5 and 2.7 Hz, H-16), 4.13 (d, *J* = 11.8 Hz, H-18α), 3.99 (dd, *J* = 15.7 and 8.0 Hz, H-15α), 3.94 (d, *J* = 10.5 Hz, H-19α), 3.78 (d, *J* = 11.8 Hz, H-18β), 3.55 (d, *J* = 10.5 Hz, H-19β), 3.40 (dd, *J* = 16.2 and 8.0 Hz, H-15β), 3.32/3.29 (s, α-MeO/β-MeO), 2.24–1.99 (m, H-14), 2.22 (m, H-13), 2.22–1.12 (m, H-3), 2.18 (m, H-7), 1.60 (m, H-2), 1.51 (m, H-8), 1.45 (m, H-9), 1.43–1.22 (m, H-1), 1.19 (m, H-11), 1.19 (m, H-12), 0.83 (d, *J* = 6.4 Hz, H-17), and 0.77 (s, H-20). ^13^C NMR (CD_3_OD, 75 MHz): δ 146.5 (C-5), 128.8 (C-6), 106.6/107.0 (C-16a/C-16b), 73.2/73.6 (C-15a/C-15b), 65.7 (C-19), 64.5 (C-18), 54.7/55.2 (α-MeO/β-MeO), 47.5 (C-9), 44.0 (C-4), 40.1 (C-14), 39.7 (C-10), 38.8 (C-13), 38.4 (C-1), 37.6 (C-8), 32.2 (C-3), 28.4/28.0 (C-12a/C-12b), 27.8/27.3 (C-11a/C-11b), 27.3 (C-7), 19.3 (C-20), 18.4 (C-2), and 16.2 (C-17). 

*Gaudichaudol C* (**2**). White amorphous solid. ESI-LRMS (negative mode) *m/z* 485 [M − H]^−^. ^1^H NMR (CD_3_OD, 300 MHz): δ 7.60 (d, *J* = 15.9, H-7′), 7.44 (d, *J* = 8.6, H-1′/H-5′), 6.80 (d, *J* = 8.6, H-2′/H-4′), 6.30 (d, *J* = 15.9, H-8′), 5.67 (t, *J =* 7.3 Hz, H-6), 4.12 (d, *J* = 5.8 Hz, H-16), 4.11 (d, *J* = 11.7 Hz, H-18α), 3.95 (d, *J* = 10.7 Hz, H-19α), 3.80 (d, *J* = 11.7 Hz, H-18β), 3.62 (t, *J* = 6.7 Hz, H-15), 3.56 (d, *J* = 10.7 Hz, H-19β), 3.30 (m, H-13), 2.22–1.13 (m, H-3), 2.19 (m, H-7), 2.18 (m, H-11), 1.61 (m, H-2), 1.51 (m, H-8), 1.45 (m, H-9), 1.44 (m, H-14), 1.39 (m, H-12), 1.20 (m, H-1), 0.82 (d, *J* = 6.4, H-17), and 0.77 (s, H-20). ^13^C NMR (CD_3_OD, 75 MHz): δ 169.2 (C-9′), 161.3 (C-3′), 146.5 (C-5), 146.5 (C-6), 131.1 (C-1′/C-5′), 128.8 (C-6), 127.0 (C-6′), 116.8 (C-2′/C-4′), 115.1 (C-8′), 67.5 (C-16), 65.2 (C-19), 64.5 (C-18), 59.8 (C-15), 49.8 (C-13), 47.6 (C-9), 44.0 (C-4), 39.8 (C-10), 37.6 (C-8), 35.9 (C-1), 35.2 (C-14), 32.2 (C-3), 28.0 (C-12), 27.3 (C-7), 24.8 (C-11), 19.5 (C-20), 18.4 (C-2), and 16.3 (C-17).

Ent-*kaur-16-en-19-oic acid* (**3**). White amorphous solid. ESI-HR-MS (negative mode): *m/z* 301.2191 [M − H]^−^. ^1^H NMR (CDCl_3_, 500 MHz): δ 4.81 (s, H-17a), δ 4.75 (s, H-17b), 2.64 (s, H-13), 1.24 (s, H-18), and 0.95 (s, H-20). ^13^C NMR (CDCl_3_, 125 MHz): δ 183.6 (C-19), 155.8 (C-16), 102.9 (C-17), 56.9 (C-5), 55.1 (C-9), 48.8 (C-15), 44.1 (C-8), 43.7 (C-4), 41.2 (C-13), 40.6 (C-1), 39.6 (C-10), 39.5 (C-7), 37.8 (C-3), 37.7 (C-14), 33.0 (C-12), 28.9 (C-18), 21.8 (C-6), 19.0 (C-2), 18.3 (C-11), and 15.5 (C-20).

*Hispidulin* (**4**). Yellow amorphous solid. ESI-LRMS (negative mode) *m/z* 299 [M − H]^−^. ^1^H NMR (CD_3_OD, 300 MHz): δ 7.81 (d, *J* = 8.9 Hz, H-2′/H-6′), 6.88 (d, *J* = 8.9 Hz, H-3′/H-5′), 6.55 (s, H-3), 6.51 (s, H-8), and 3.83 (s, MeO). ^13^C NMR (CD_3_OD, 75 MHz): δ 184.2 (C-4), 166.3 (C-2), 162.8 (C-4′), 154.7 (C-5), 154.0 (C-9), 131.8 (C-6), 129.4 (C-2′/C-6′), 123.2 (C-1′), 117.0 (C-3′/C-5′), 106.7 (C-10), 103.3 (C-3), 95.4 (C-8), and 60.9 (MeO).

*Eupafolin* (**5**). Yellow amorphous solid. ESI-LRMS (negative mode) *m/z* 315 [M − H]^−^. ^1^H NMR (CD_3_OD, 300 MHz): δ 7.35 (dd, *J* = 8.9 and 2.2 Hz, H-6′), 7.34 (d, *J* = 2.2 Hz, H-2′), 6.87 (d, *J* = 8.9 Hz, H-5′), 6.50 (s, H-3), 6.50 (s, H-8), and 3.83 (s, MeO). ^13^C NMR (CD_3_OD, 75 MHz): δ 184.2 (C-4), 166.4 (C-2), 158.9 (C-7), 154.7 (C-5), 154.7 (C-9), 151.0 (C-4′), 147.1 (C-3′), 132.9 (C-6), 123.7 (C-1′), 120.3 (C-6′), 116.8 (C-5′), 114.1 (C-2′), 105.7 (C-10), 103.4 (C-3), 95.2 (C-8), and 60.9 (MeO).

*3,4-Di-O-caffeoylquinic acid* (**6**). Yellow amorphous solid. ESI-LRMS (negative mode) *m/z* 515 [M − H]^−^. ^1^H NMR (DMSO-*d*_6_, 300 MHz): δ 7.49 (d, *J* = 15.9, H-7′), 7.47 (d, *J* = 15.9, H-7″), 7.06 (s, H-2′/H-2″), 6.98 (s, H-6′/H-6″), 6.78 (d, *J =* 8.0 Hz, H-5′/H-5″), 6.27 (d, *J* = 15.9 Hz, H-8′), 6.18 (d, *J* = 15.9 Hz, H-8″), 5.41 (s, H-3), 5.06 (d, *J* = 6.7 Hz, H-4), 4.18 (s, H-5), 2.20 (d, *J* = 11.9 Hz, H-2), 2.09 (d, *J* = 11.9 Hz, H-6a), and 1.92 (d, *J* = 6.1 Hz, H-6b). ^13^C NMR (DMSO-*d*_6_, 75 MHz): δ 176.6 (C-7), 166.4 (C-9‘), 166.1 (C-9‘‘), 148.8 (C-3/C-3‘‘), 145.9 (C-4‘/C-4‘‘), 145.9 (C-7‘/C-7‘‘), 125.7 (C-1′/C-1‘’), 121.3 (C-6‘), 121.8 (C-6‘‘), 116.1 (C-2′), 116.0 (C-2‘‘), 115.1 (C-5‘), 115.0 (C-5‘‘), 114.4 (C-8‘), 114.0 (C-8‘‘), 74.4 (C-1), 73.4 (C-4), 70.0 (C-3), 68.1 (C-5), 47.6 (C-9), 44.0 (C-4), 39.8 (C-10), 37.8 (C-2), 37.6 (C-6), 37.6 (C-8), 35.8 (C-1), 34.6 (C-14), 32.2 (C-3), and 28.0 (C-5‘‘).

*3,5-Di-O-caffeoylquinic acid* (**7**). Yellow amorphous solid. ESI-LRMS (negative mode) *m/z* 515 [M − H]^−^. ^1^H NMR (DMSO-*d*_6_, 300 MHz): δ 7.48 (d, *J* = 15.9, H-7′), 7.45 (d, *J* = 15.9, H-7″), 7.06 (s, H-2′/H-2″), 7.09 (d, *J* = 8.0 H-6′/H-6″), 6.77 (m, H-5′/H-5″), 6.27 (d, *J* = 15.9 Hz, H-8′), 6.14 (d, *J* = 15.9 Hz, H-8″), 5.78 (s, H-3), 5.41 (s, H-5), 3.87 (s, H-4), 2.21 (m, H-2/H-6), and 2.02 (m, H-2/H-6). ^13^C NMR (DMSO-*d*_6_, 75 MHz): δ 176.6 (C-7), 166.4 (C-9‘), 166.1 (C-9‘‘), 148.8 (C-3/C-3‘‘), 145.9 (C-4‘/C-4‘‘), 145.9 (C-7‘/C-7‘‘), 125.7 (C-1′/C-1‘’), 121.9 (C-6‘), 121.3 (C-6‘‘), 116.1 (C-2′), 116.1 (C-2‘‘), 115.1 (C-5‘), 115.1 (C-5″), 114.4 (C-8‘), 114.0 (C-8‘‘), 74.4 (C-1), 73.4 (C-4), 70.0 (C-3), 68.1 (C-5), 47.6 (C-9), 44.0 (C-4), 39.8 (C-10), 37.8 (C-2), 37.6 (C-6), 37.6 (C-8), 35.9 (C-1), 35.2 (C-14), 32.2 (C-3), and 28.0 (C-5‘‘).

*4,5-Di-O-caffeoylquinic acid* (**8**). Yellow amorphous solid. ESI-LRMS (negative mode) *m/z* 515 [M − H]^−^. ^1^H NMR (DMSO-*d*_6_, 300 MHz): δ 7.51 (d, *J* = 15.9, H-7′), 7.52 (d, *J* = 15.9, H-7″), 7.09 (s, H-2′/H-2″), 6.97 (s, H-6′/H6″), 6.82 (d, *J =* 8.0 Hz, H-5′/H-5″), 6.33 (d, *J* = 15.9 Hz, H-8′), 6.24 (d, *J* = 15.9 Hz, H-8″), 5.34 (s, H-5), 5.29 (s, H-4), 3.96 (s, H-3), 2.25 (s, H-2/H-6), and 2.15 (s, H-2/H-6). ^13^C NMR (DMSO-*d*_6_, 75 MHz): δ 177.1 (C-7), 166.6 (C-9′), 166.5 (C-9″), 148.8 (C-3), 148.7 (C-3‘‘), 145.9 (C-4‘/C-4‘‘), 146.1 (C-7′), 145.8 (C-7″), 125.8 (C-1′/C-1‘’), 121.5 (C-6′), 121.3 (C-6″), 116.1 (C-2′/C-2″), 116.0 (C-5‘), 115.1 (C-5″), 114.9 (C-8′), 114.6 (C-8″), 73.4 (C-1), 71.6 (C-4), 70.2 (C-5), 68.1 (C-3), 36.7 (C-2), and 35.4 (C-6).

### 3.6. Animal Models, Mammalian Cells, and Parasites Maintenance

The experimental animals were sourced from the animal breeding facility located at Instituto Adolfo Lutz in São Paulo State, Brazil. Peritoneal macrophages were extracted from female BALB/c mice for intracellular experiments, while male BALB/c mice were utilized to sustain *T. cruzi*. These animals were provided unlimited access to both water and food, residing in sterilized enclosures within a meticulously regulated environment. All procedures were previously approved by the Animal Ethics Commission of Instituto Adolfo Lutz.

NCTC cells (clone 929, ATCC) were cultured in RPMI-1640 supplemented with 10% of fetal bovine serum (FBS), at 37 °C within a 5% CO_2_-humidified incubator. To collect peritoneal macrophages, the abdominal cavity of female BALB/c mice was rinsed with an RPMI-1640 medium, containing 10% FBS. These macrophages were then cultured overnight at 37 °C in a 5% CO_2_-humidified incubator. *Trypanosoma cruzi* trypomastigotes (Y strain) were maintained within Rhesus monkey kidney epithelial cells (LLC-MK2) cells using an RPMI-1640 medium supplemented with 2% FBS, and the incubation took place at 37 °C with 5% CO_2_ and humidity.

### 3.7. Determination of Activity (EC_50_) of Compounds **1**–**8** against Trypomastigote and Amastigote Forms of Trypanosoma cruzi

Tests of the activity from compounds **1**–**8** against the trypomastigote form of *T. cruzi* was performed, according to procedures previously reported [24]. Trypomastigotes, in a concentration of 1 × 10^6^ parasites/well, were seeded in 96-well plates and incubated with the tested compounds, alongside the standard drug benznidazole, in serial dilutions ranging from 1.7 to 150 μM, for 24 h at 37 °C in a 5% CO_2_ incubator. Subsequently, 20 μL of 10% resazurin was added, in order to determine cell viability. Absorbance was read in a plate spectrofluorometer Filter Max F5 Multi-Mode Microplate Reader at 570 nm. Untreated cells were used as negative controls, and cells treated with benznidazole were used as positive controls. The 50% effective concentration (EC_50_) against trypomastigote forms was determined by the regression of a sigmoid dose–response curve expressed in μM.

The EC_50_ values for compounds **1**–**8** against intracellular amastigotes of *T. cruzi* were determined within infected macrophages. Macrophages, obtained as detailed in item 4.9, were seeded onto a 16-well chamber slide (NUNC plate, Thermo Fisher, Waltham, MA, USA) at a density of 1 × 10^5^ cells per well. Subsequently, these cell cultures were incubated overnight at 37 °C in a humidified incubator containing 5% CO_2_. Trypomastigotes, previously derived from infected LLC-MK2 cells, underwent washing in an RPMI-1640 medium, were quantified, and were then employed to infect the macrophages at a concentration of 1 × 10^6^ trypomastigotes per well. Following a 2 h infection period, any non-internalized trypomastigotes were eliminated by rinsing with an RPMI medium. The compounds were subsequently exposed to the infected macrophages for 48 h at 37 °C within a 5% CO_2_ environment. A range of serial dilutions (ranging from 30.00 to 1.87 μM) were used for the incubation of compounds, and benznidazole was utilized as the standard drug. At the final stage of the experiment, the chamber slides were fixed using MeOH and stained with Giemsa before being assessed under a light microscope (Thermo EVOS M5000). 

### 3.8. Determination of Cytotoxicity (CC_50_) against Mammalian Cells of Compounds **1**–**8**

The determination of CC_50_ (50% cytotoxic concentration) values for compounds **1**–**8** was conducted using NCTC cells (L929 clone). These cells were seeded at a density of 6 × 10^4^ cells per well in 96-well plates (Thermo Fisher). The cells were then exposed to a series of compound dilutions, ranging from 200 to 1.56 µM, and incubated for 48 h at 37 °C within a 5% CO_2_-equipped incubator. The viability of the cells and CC_50_ values were determined using 3-(4,5-dimethylthiazol-2-yl)-2,5-diphenyltetrazolium bromide (MTT) assay. Subsequently, the absorbance was measured at 570 nm using the FilterMax F5 instrument from Molecular Devices. The selectivity index (SI) was determined as the ratio between the CC_50_ against NCTC cells and the EC_50_ against trypomastigotes or amastigotes.

### 3.9. Statistical Analysis 

The CC_50_ and EC_50_ values were derived through the analysis of sigmoidal dose–response curves using GraphPad Prism 6.0 software. The samples were subjected to duplicate testing, and the experiments were replicated a minimum of two times. 

## 4. Conclusions

In this work, three diterpenes (**1**–**3**), two flavones (**4** and **5**), and a mixture of three di-*O*-caffeoylquinic acids (**6**–**8**) were isolated from aerial parts of *B. sphenophylla*, by means of high-performance countercurrent chromatography (HPCCC), using specifically target-adjusted HemWat biphasic solvent systems. This work advances the chemosystematics knowledge of the *Baccharis* genus and shows an application of HPCCC methodology for the isolation of anti*-T. cruzi* compounds **1**–**8**. As result, compound **2** displayed potent activity against trypomastigotes while compound **5** exhibited intense activity against the clinically relevant amastigote forms of *T. cruzi*. These results allowed for the development of molecular prototypes for the development of new drugs for the treatment of Chagas disease. 

## Figures and Tables

**Table 1:** Activity against amastigote forms of *T. cruzi* and cytotoxicity against mammalian (NCTC) cells of compounds **1**–**8**, isolated from MeOH extract from aerial parts of *B. sphenophylla*.

Compound	*T. cruzi* Trypomastigote EC_50_ (μM) ± SD	*T. cruzi* Amastigote EC_50_ (μM) ± SD	NCTC Mammalian Cell CC_50_ (μM) ± SD	*T. cruzi* Trypomastigote SI	*T. cruzi* Amastigote SI
1	20.1 ± 1.5	NA	>200	>10.0	-
2	2.9 ± 1.5	NA	>200	>68.7	-
3	10.6 ± 4.4	NA	>200	>18.8	-
4	NA	12.8 ± 0.3	>200	-	>12.6
5	NA	2.7 ± 0.2	>200	-	>74.1
6–8	NA	24.9 ± 1.3	>200	-	>8.0
Benznidazole	18.7 ± 4.1	5.5 ± 2.2	>200	>10.7	>36.4

EC_50_—50% effective concentration; CC_50_—50% cytotoxic concentration; SI—selectivity index; NA—not active; SD—standard deviation; samples were tested in duplicate and the assays were repeated at least twice.

## Data Availability

The data presented in this study are available in the Supplementary Materials.

## Supplementary Materials

The following supporting information can be downloaded at: https://www.mdpi.com/article/10.3390/molecules29010212/s1. Figure S1: ^1^H NMR spectrum of compound **1** (δ, 300 MHz, CD_3_OD). Figure S2: ^13^C NMR spectrum of compound **1** (δ, 75 MHz, CD_3_OD). Figure S3: DEPT spectrum of compounds **1** (δ, 75 MHz, CD_3_OD). Figure S4: HQSC spectrum of compound **1** (δ, 300/75 MHz, CD_3_OD). Figure S5. HMBC spectrum of compound **1** (δ, 300/75 MHz, CD_3_OD). Figure S6: NOESY spectrum of compound **1** (δ, 300 MHz, CD_3_OD). Figure S7. HR-ESI-MS of compounds **1** (positive mode). Figure S8. ^1^H NMR spectrum of compound **2** (δ, 300 MHz, CDCl_3_). Figure S9. ^13^C NMR spectrum of compound **2** (δ, 75 MHz, CDCl_3_). Figure S10. ESI-LRMS of compound **2** (negative mode). Figure S11. ^1^H NMR spectrum of compound **3** (δ, 300 MHz, CDCl_3_). Figure S12. ^13^C NMR spectrum of compound **3** (δ, 75 MHz, CDCl_3_). Figure S13. ESI-HRMS of compound **3** (negative mode). Figure S14. ^1^H NMR spectrum of compound **4** (δ, 300 MHz, CDCl_3_). Figure S15. ^13^C NMR spectrum of compound **4** (δ, 75 MHz, CDCl_3_). Figure S16. ESI-LRMS of compound **4** (negative mode). Figure S17. ^1^H NMR spectrum of compound **5** (δ, 300 MHz, CDCl_3_). Figure S18. ^13^C NMR spectrum of compound **5** (δ, 75 MHz, CDCl_3_). Figure S19. ESI-MS of compound **5** (negative mode). Figure S20. ^1^H NMR spectrum of compounds **6**–**8** (δ, 300 MHz, DMSO-d_6_). Figure S21. ^13^C NMR spectrum of compounds **6**–**8** (δ, 75 MHz, DMSO-d_6_). Figure S22. ESI-LRMS spectrum of compounds **6**–**8** (negative mode).
