# Supplementary material for: Evaluation of Anti-Trypanosoma cruzi Activity of Chemical Constituents from Baccharis sphenophylla Isolated Using High-Performance Countercurrent Chromatography
Source: Molecules. 2023 Dec 30;29(1):212. doi: 10.3390/molecules29010212 (PMC10780275; doi:10.3390/molecules29010212)
Supplement: Supplementary file 1 [file molecules-29-00212-s001.zip › molecules-2711345-supplementary.pdf]

Evaluation of anti-*Trypanosoma cruzi* activity of chemical constituents from *Baccharis sphenophylla* isolated using high-performance countercurrent chromatography

Matheus L. Silva[1], Felipe S. Sales[1], Erica V. C. Levatti[2], Guilherme M. Antar[3], Andre G. Tempone[2], João Henrique G. Lago[1,*] and Gerold Jerz[4,*]

[1]Center of Natural Sciences and Humanities, Federal University of ABC, Santo André 09210-580, Brazil

[2]Laboratory of Pathophysiology, Butantan Institute, São Paulo 05508-040, Brazil

[3]Department of Agrarian and Biological Sciences, Espirito Santo Federal University, São Mateus 29932-540, Brazil

[4]Institute of Food Chemistry, Technical University of Braunschweig, 38106, Braunschweig, Germany

*e-mail: g.jerz@tu-braunschweig.de and joao.lago@ufabc.edu.br

SUPPLEMENTARY MATERIAL

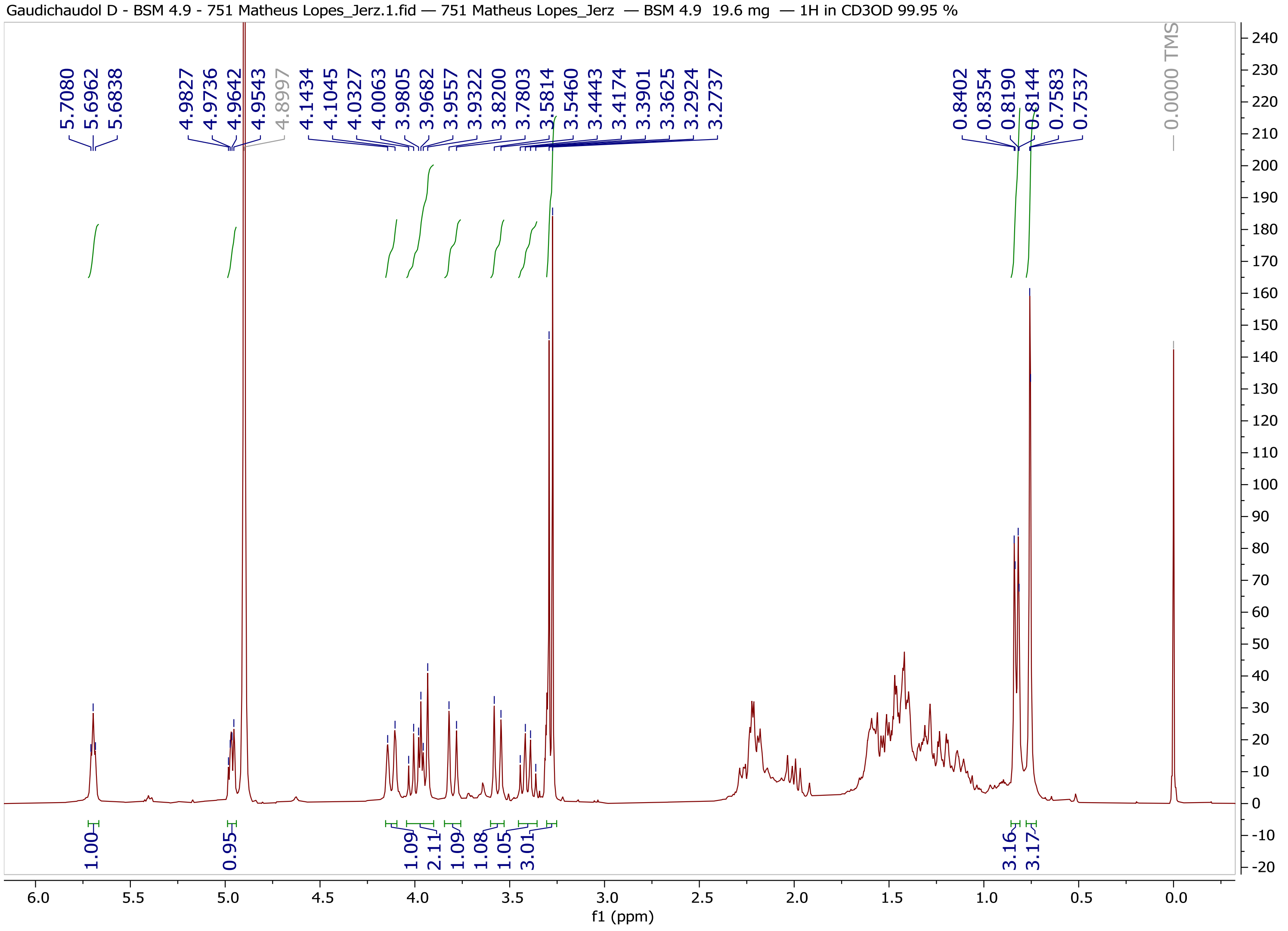

Figure S1. ^{1}H NMR spectrum of compound **1** (δ, 300 MHz, CD_3OD).

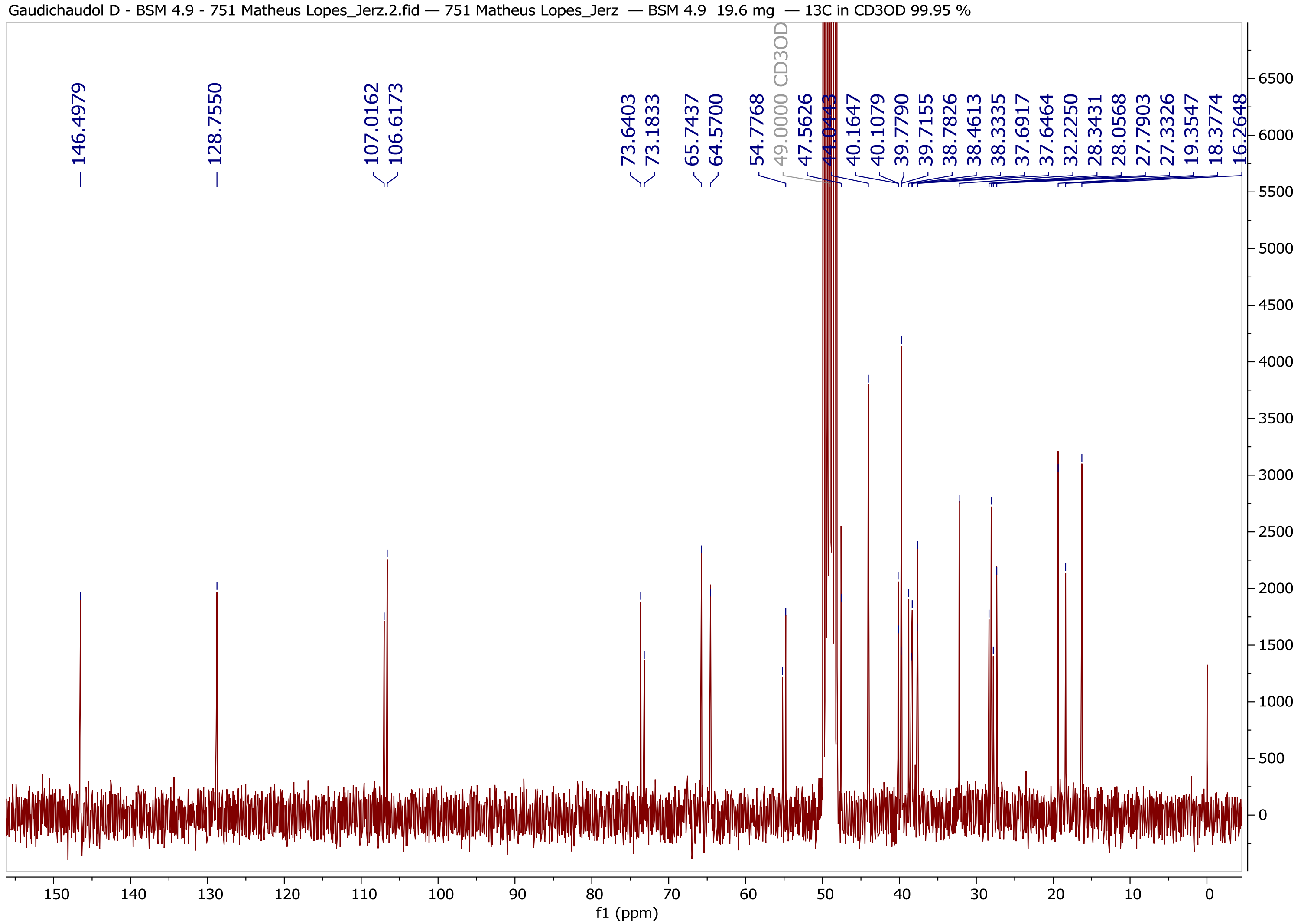

Figure S2. ^{13}C NMR spectrum of compound **1** (δ, 75 MHz, CD_3OD).

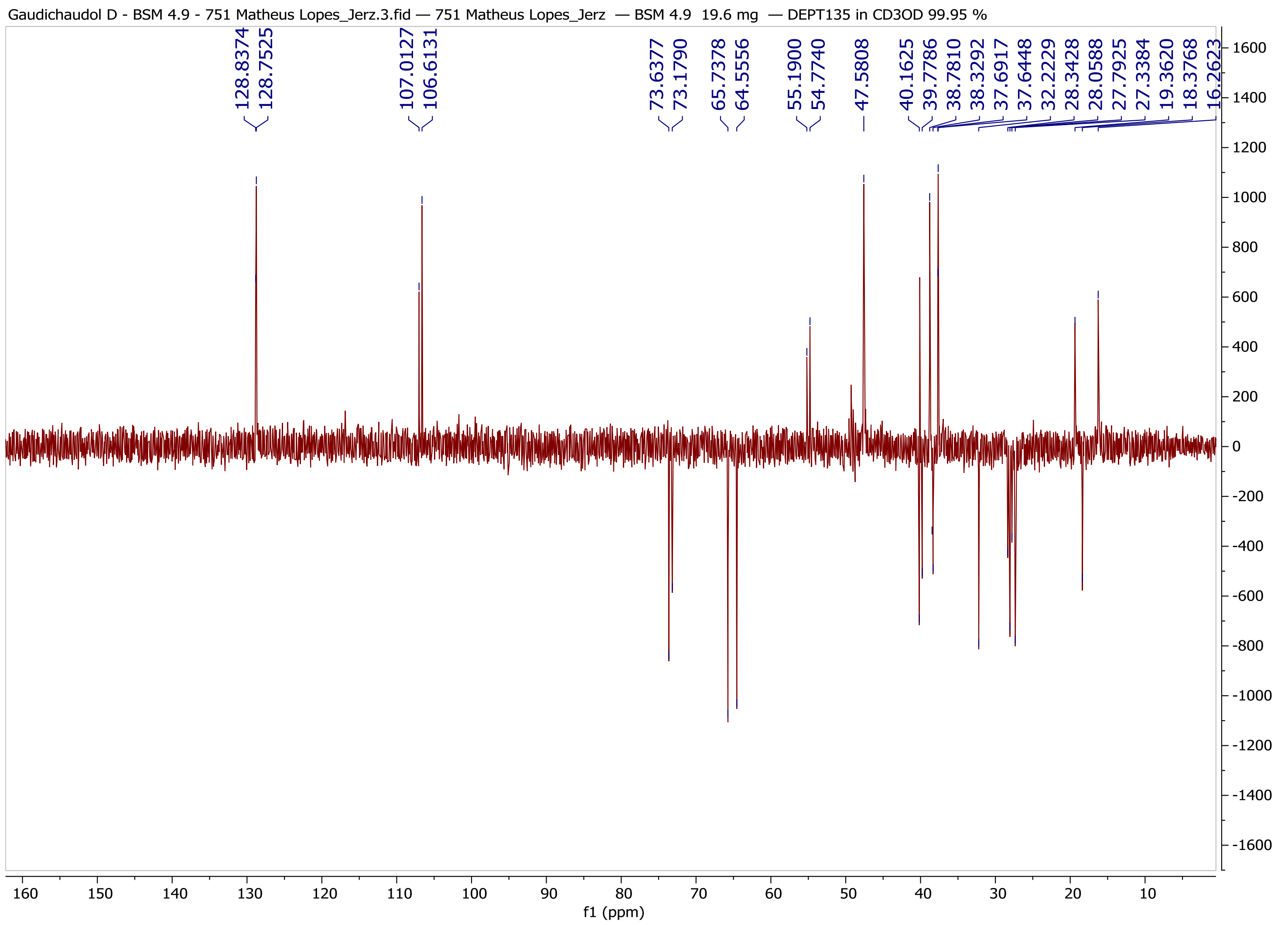

Figure S3. DEPT spectrum of compounds **1** (δ, 75 MHz, CD_3OD).

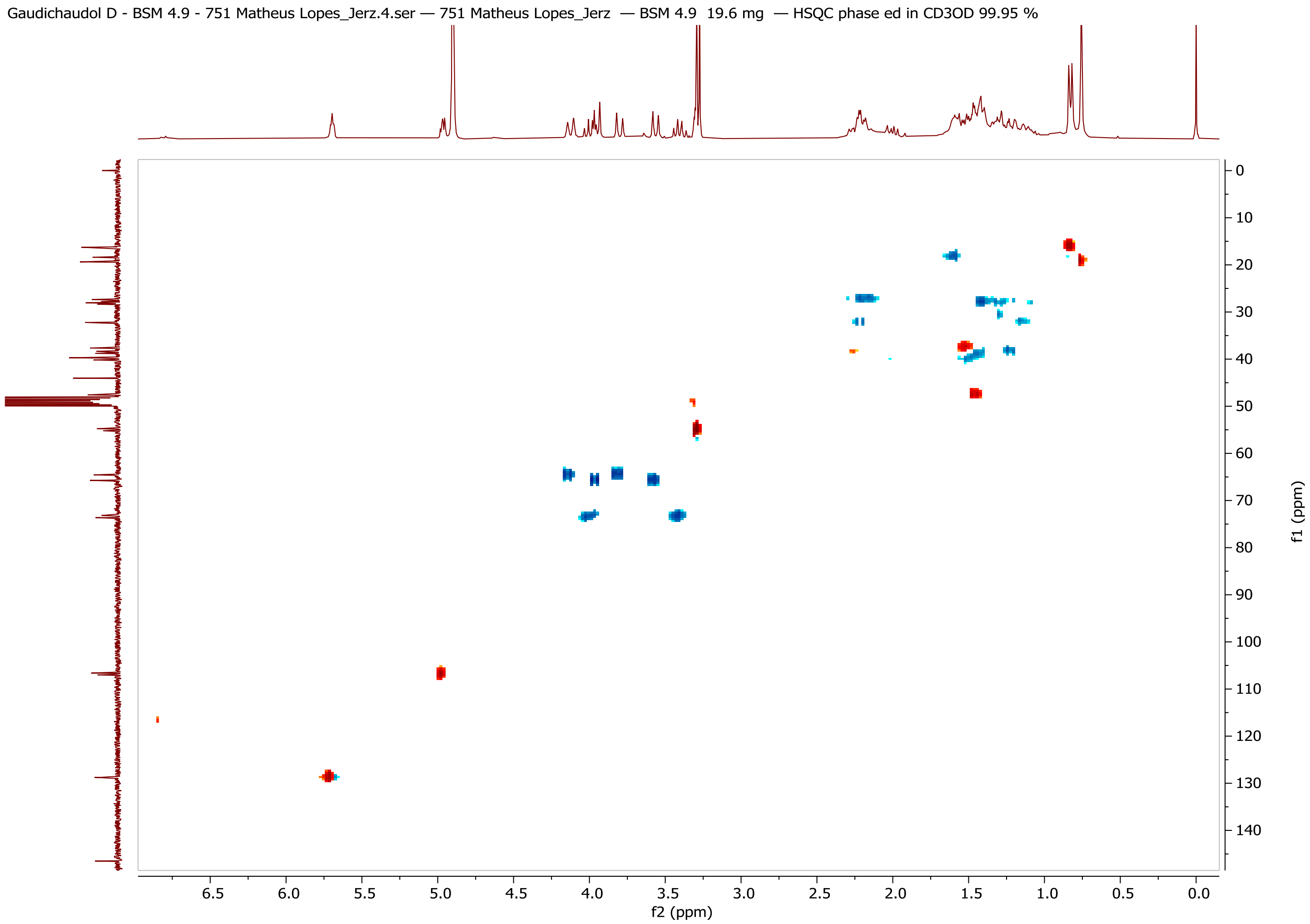

Figure S4. HQSC spectrum of compound **1** (δ, 300/75 MHz, CD_3OD).

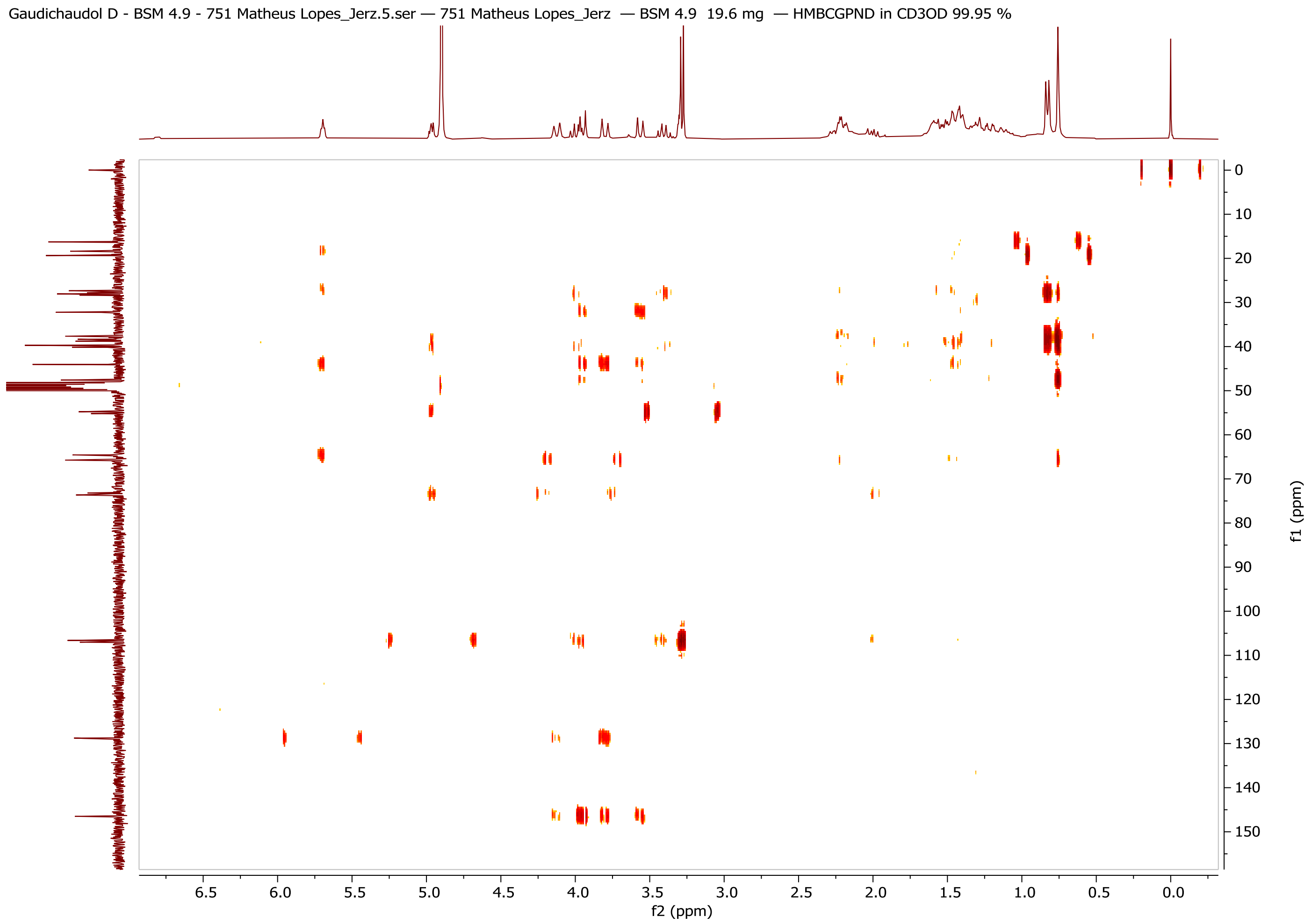

Figure S5. HMBC spectrum of compound **1** (δ, 300/75 MHz, CD_3OD).

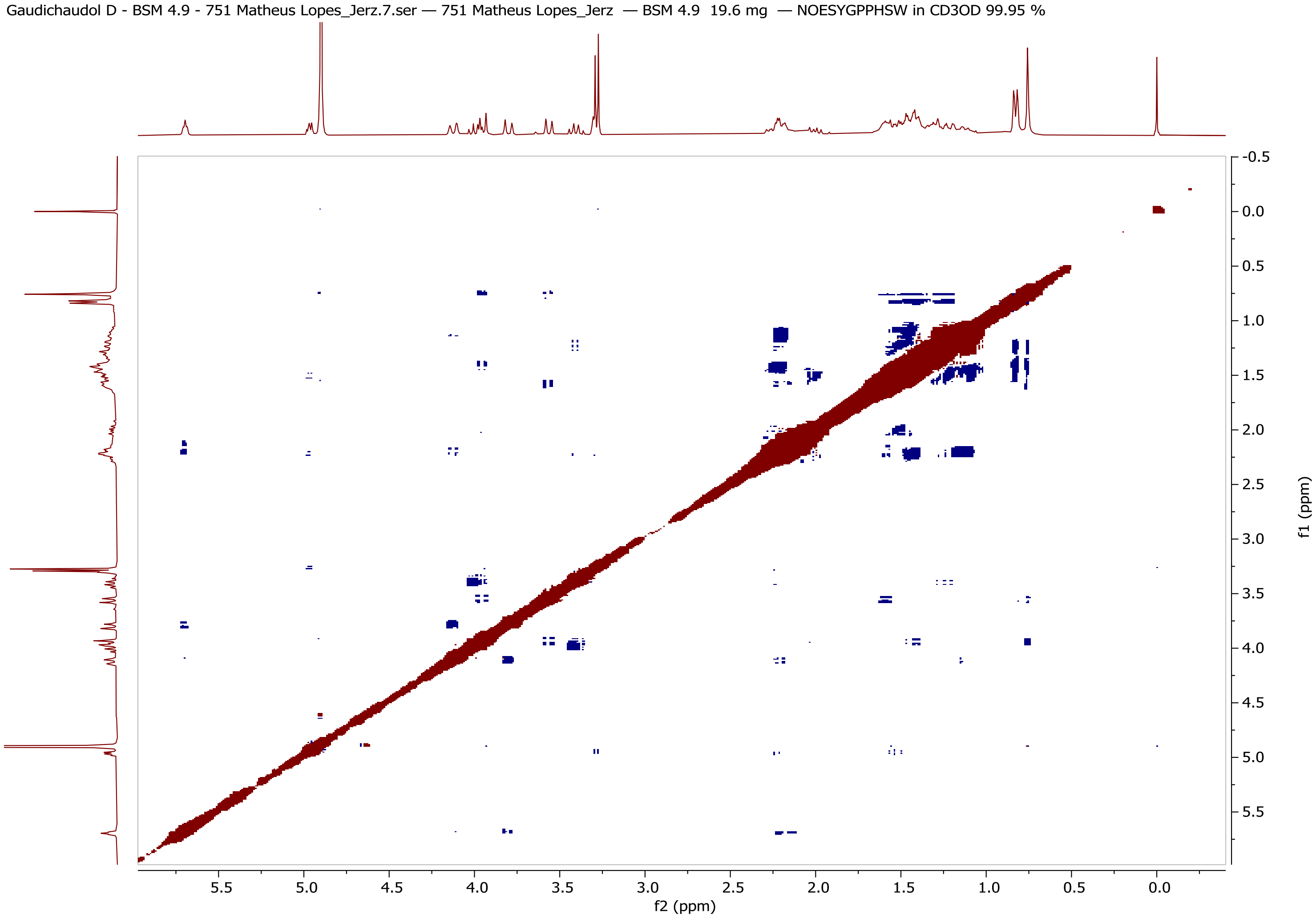

Figure S6. NOESY spectrum of compound **1** (δ, 300 MHz, CD_3OD).

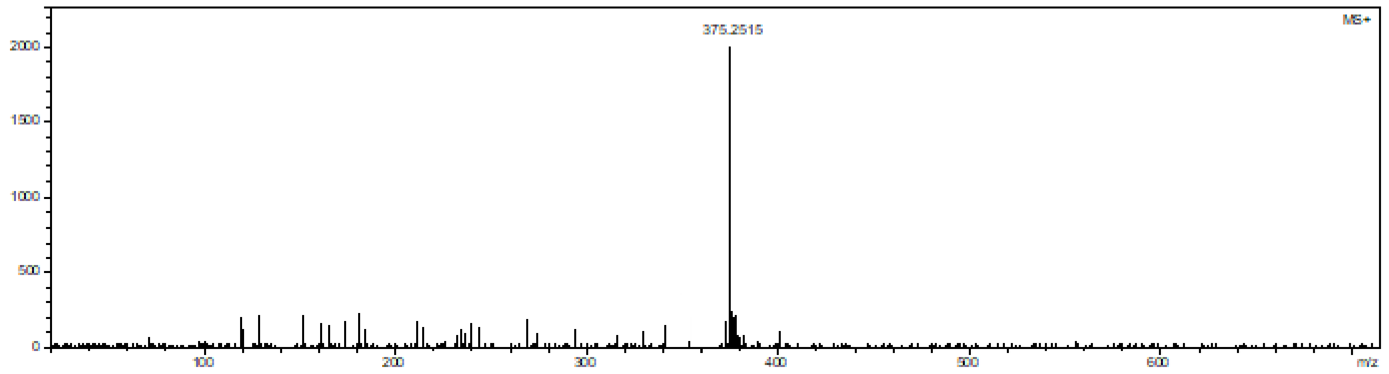

Figure S7. HR-ESI-MS of compounds **1** (positive mode).

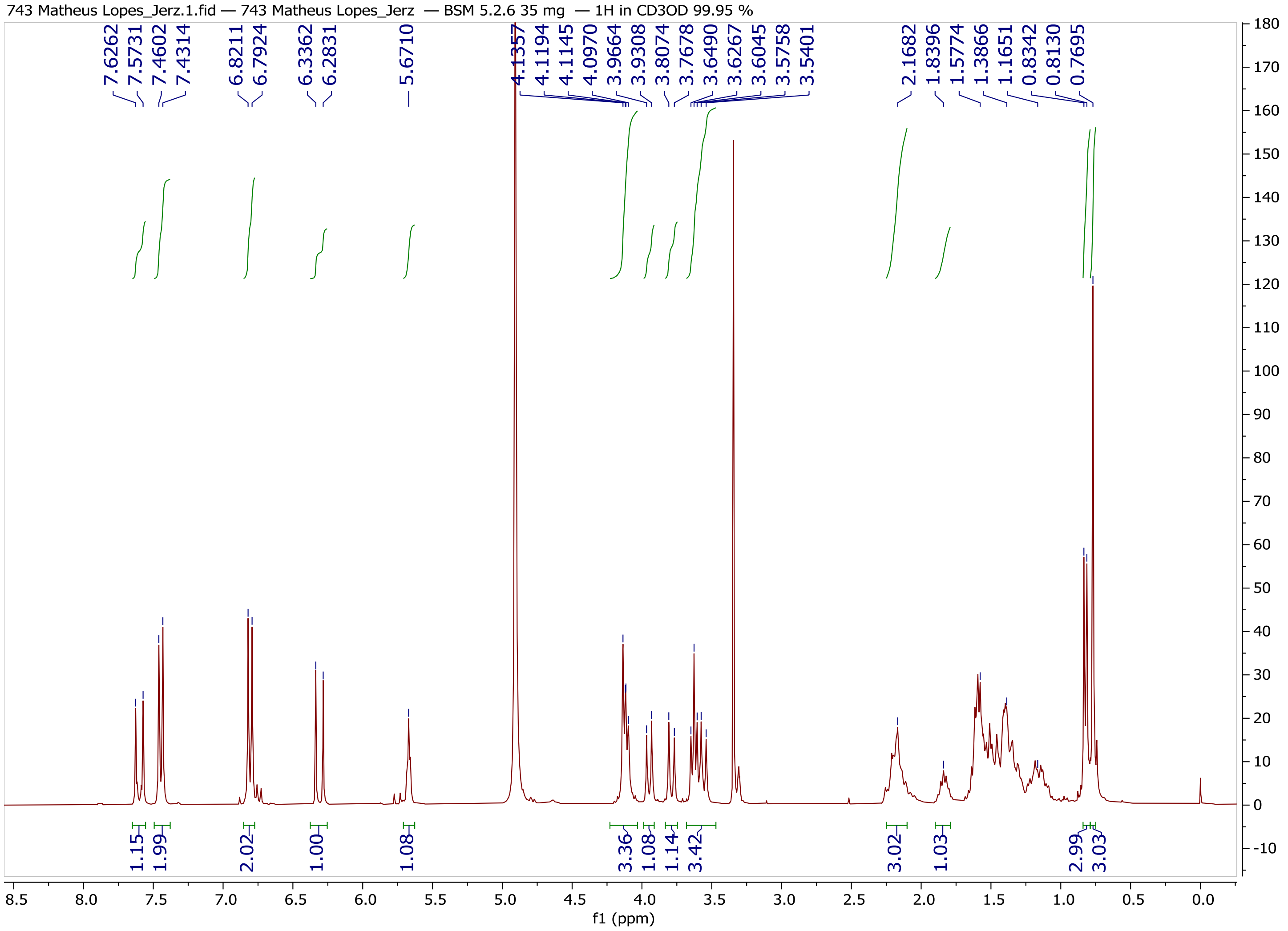

Figure S8. ^{1}H NMR spectrum of compound **2** (δ, 300 MHz, $CDCl_3$).

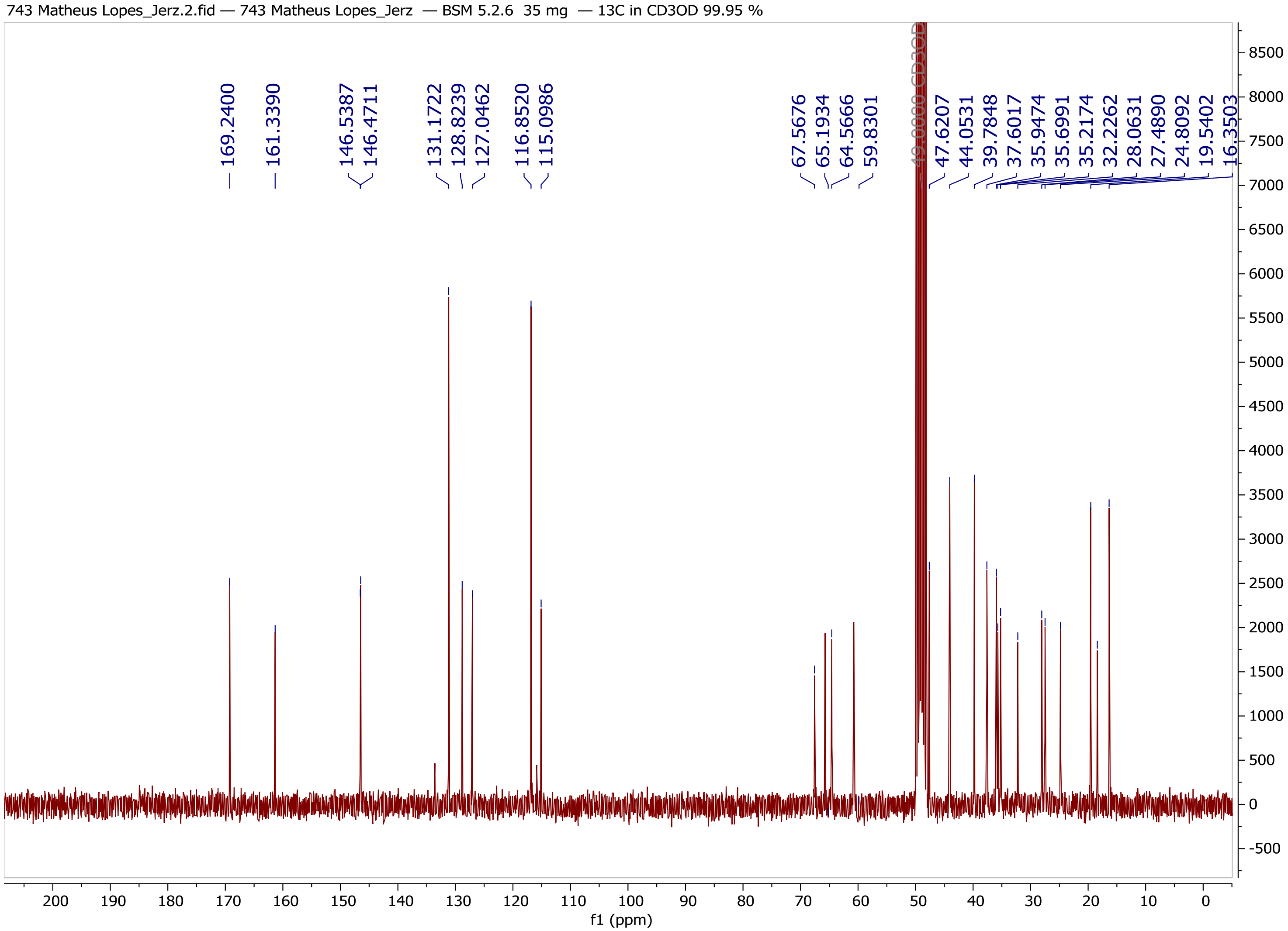

Figure S9. ^{13}C NMR spectrum of compound **2** (δ, 75 MHz, $CDCl_3$).

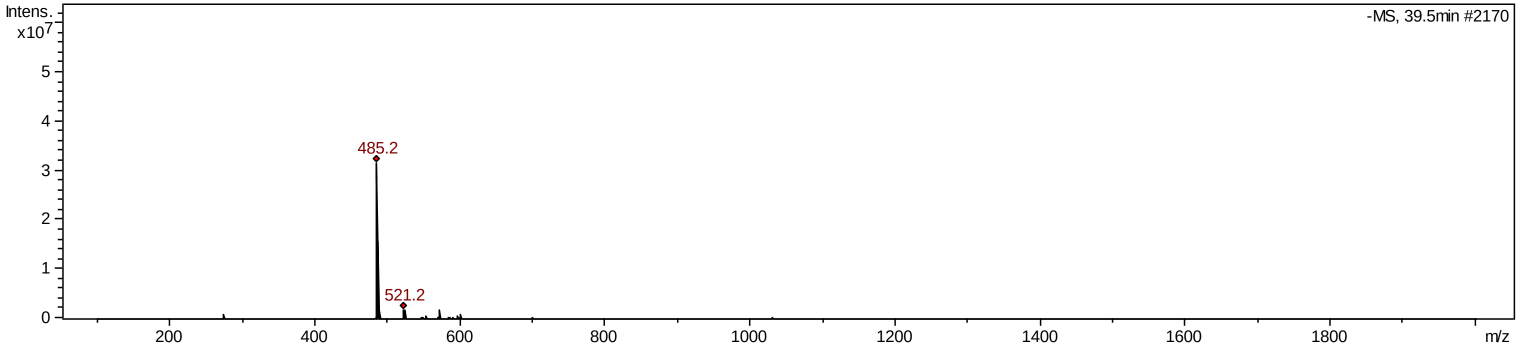

Figure S10. ESI-LRMS of compound **2** (negative mode).

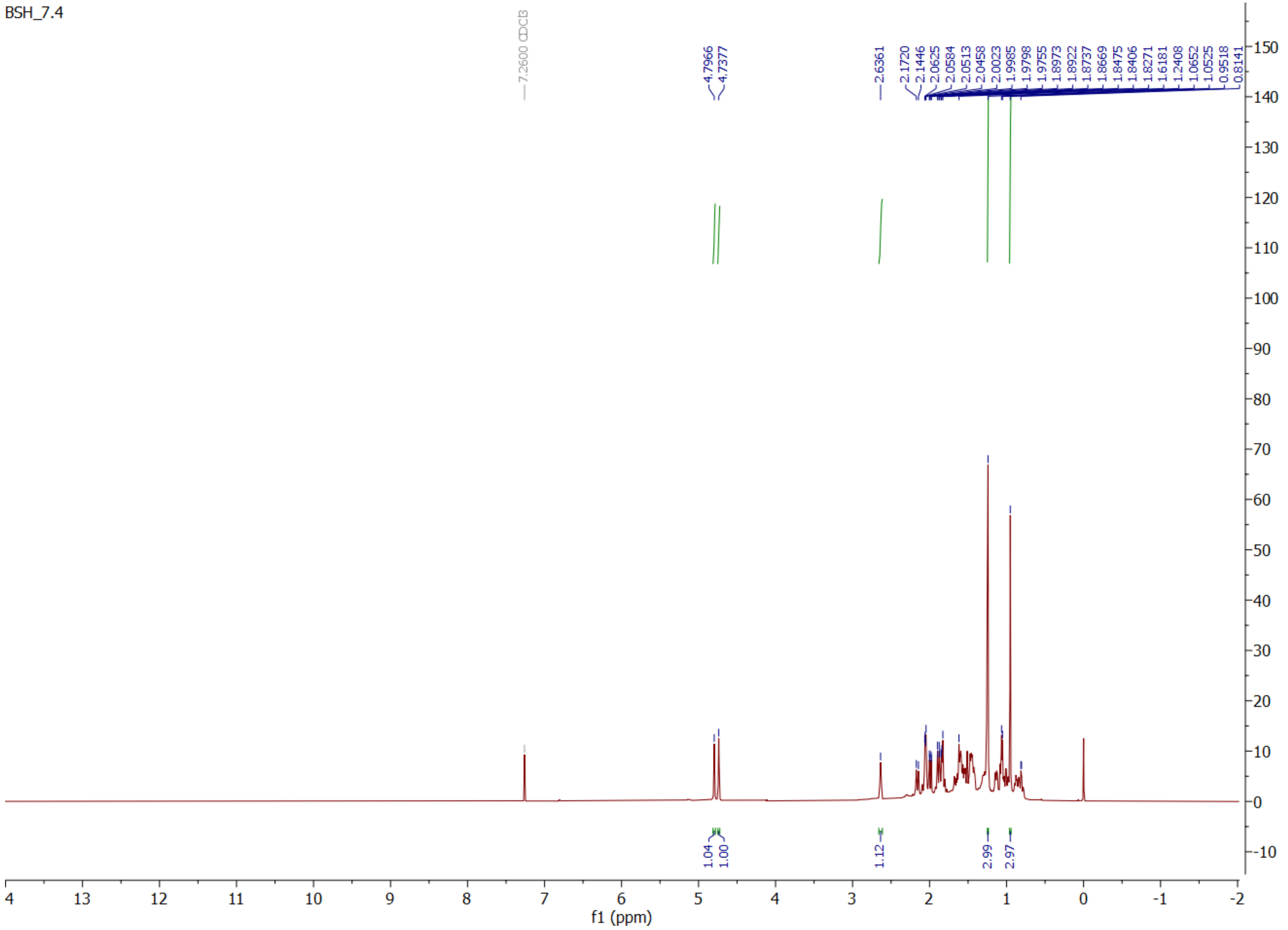

Figure S11. ^{1}H NMR spectrum of compound **3** (δ, 300 MHz, $CDCl_3$).

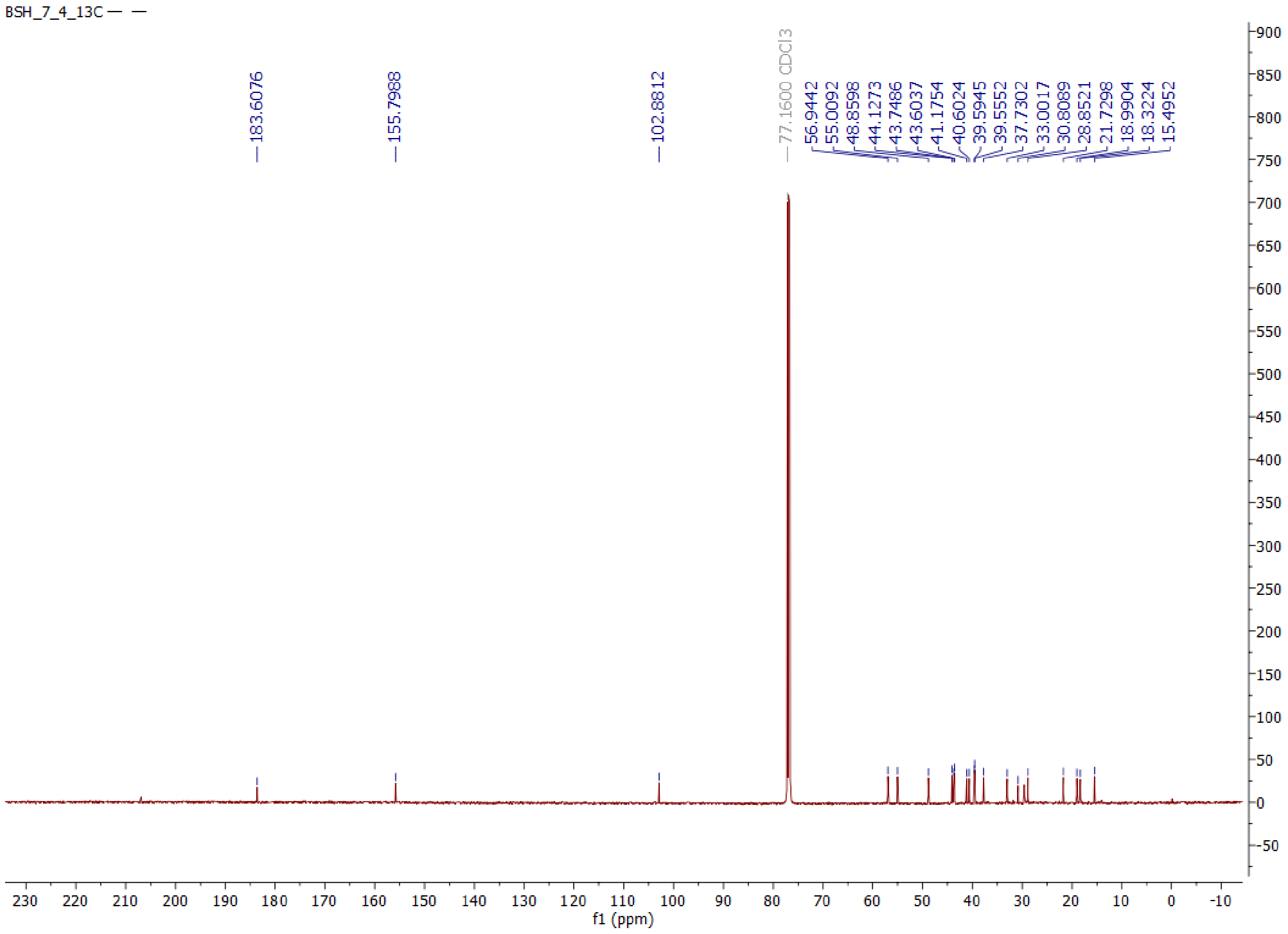

Figure S12. ^{13}C NMR spectrum of compound **3** (δ, 75 MHz, $CDCl_3$).

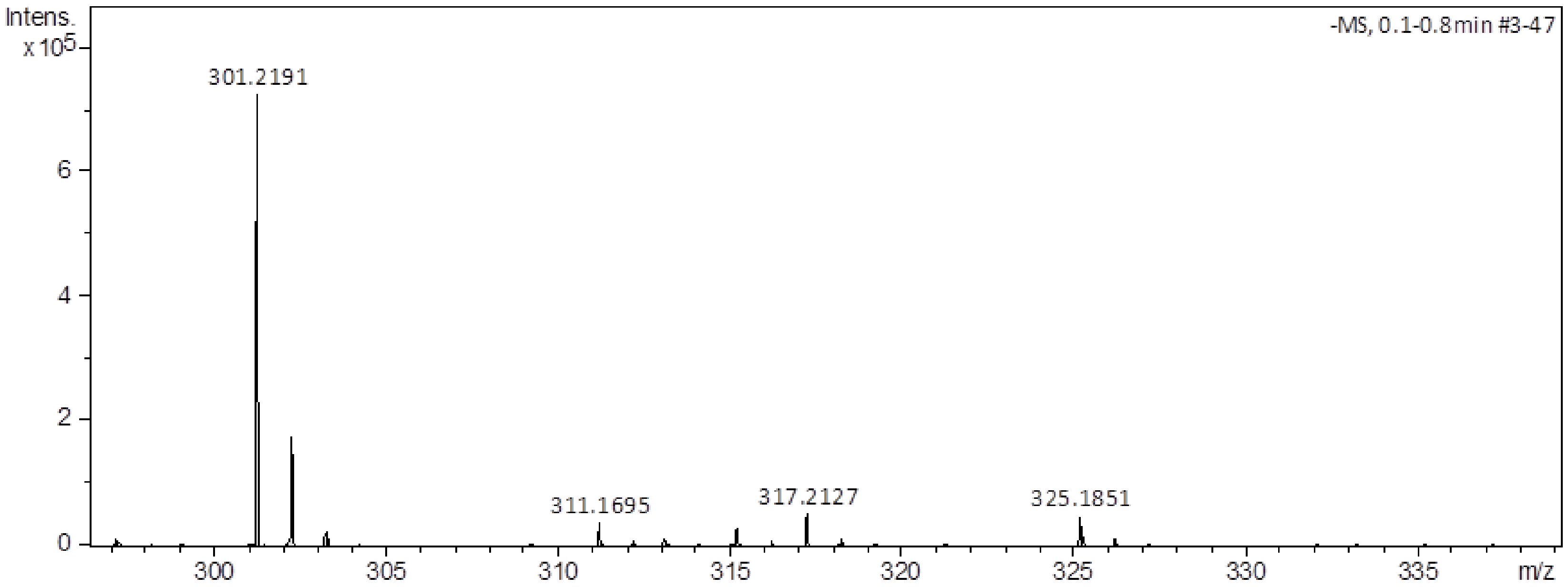

Figure S13. ESI-HRMS of compound **3** (negative mode).

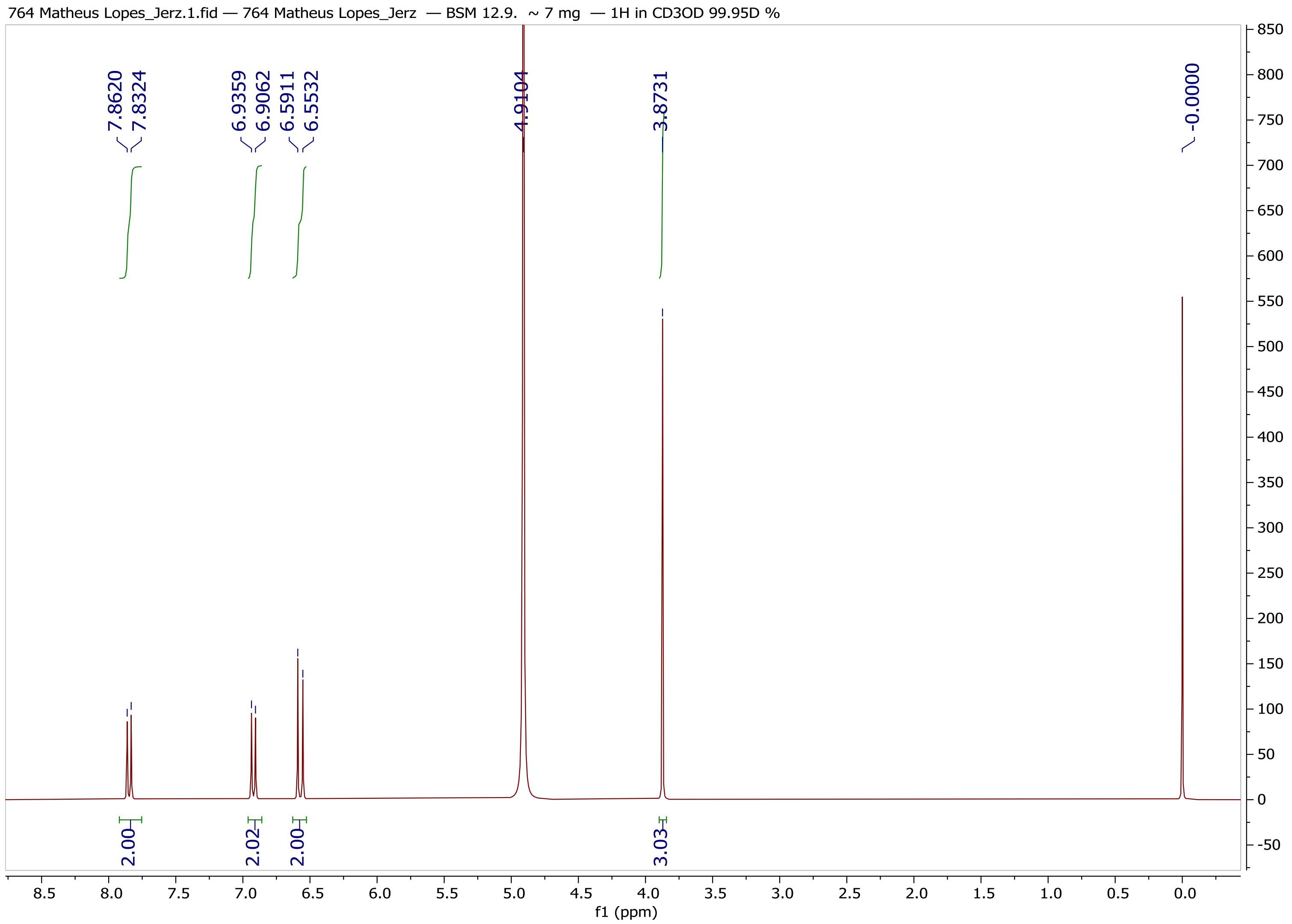

Figure S14. ^{1}H NMR spectrum of compound **4** (δ, 300 MHz, $CDCl_3$).

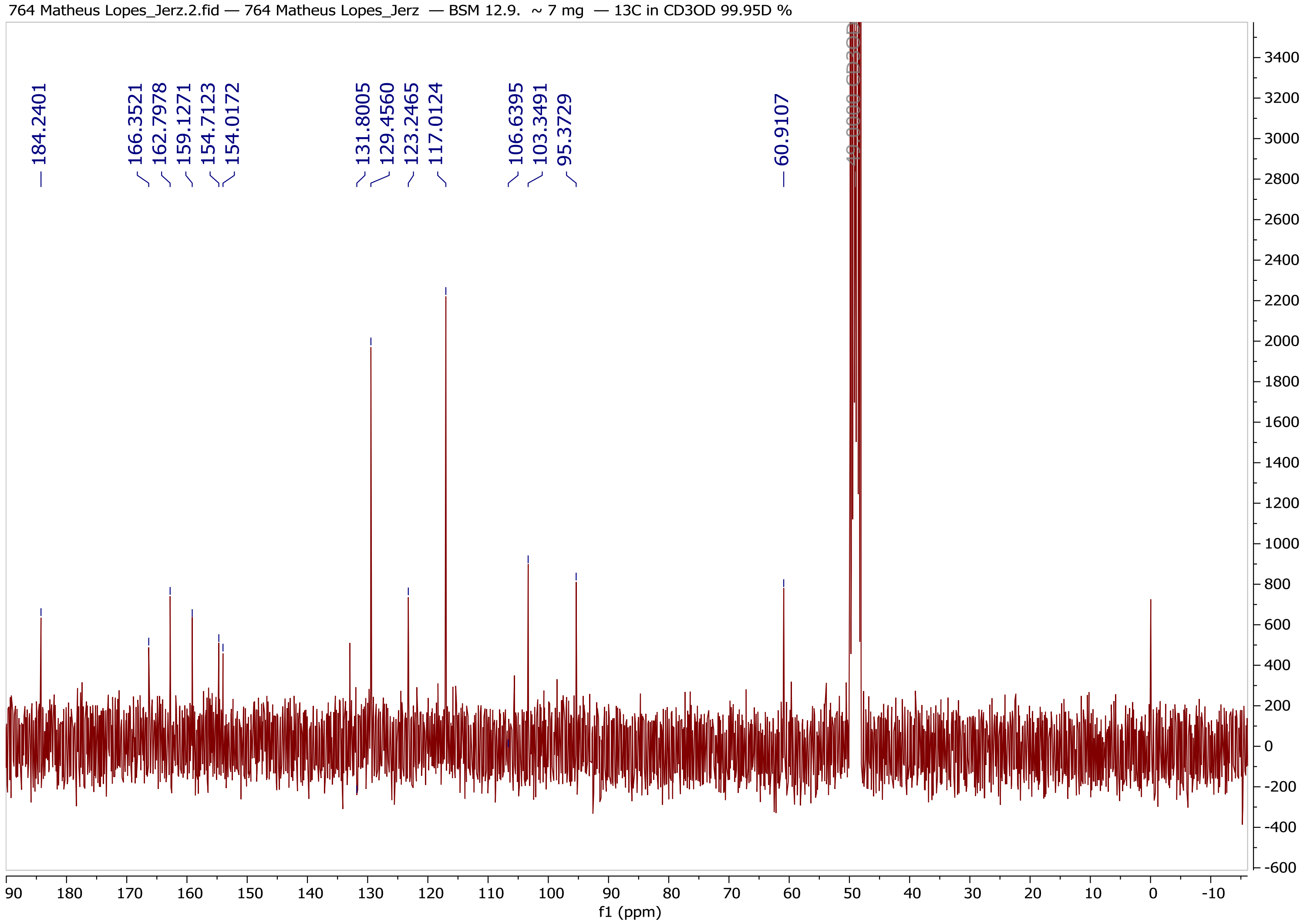

Figure S15. ^{13}C NMR spectrum of compound **4** (δ, 75 MHz, $CDCl_3$).

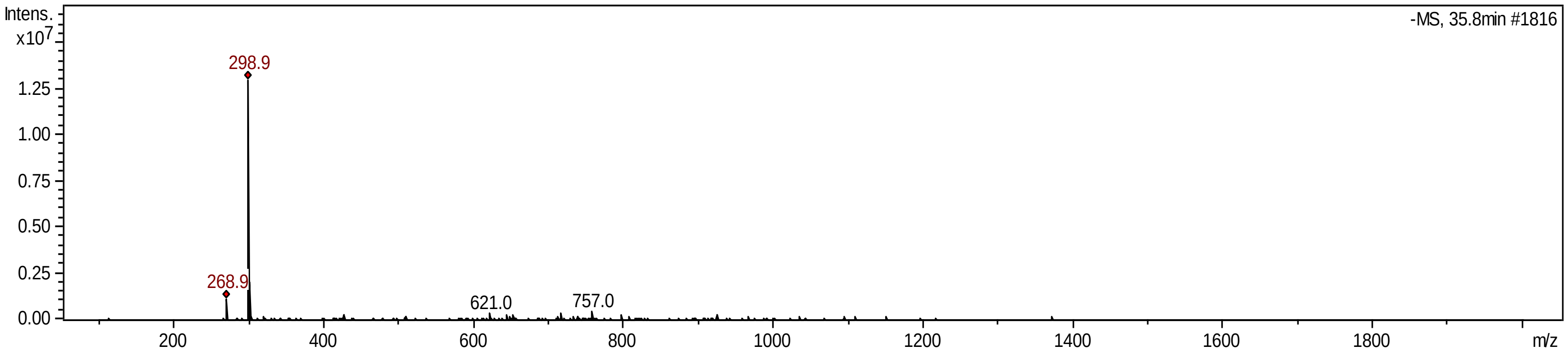

Figure S16. ESI-LRMS of compound **4** (negative mode).

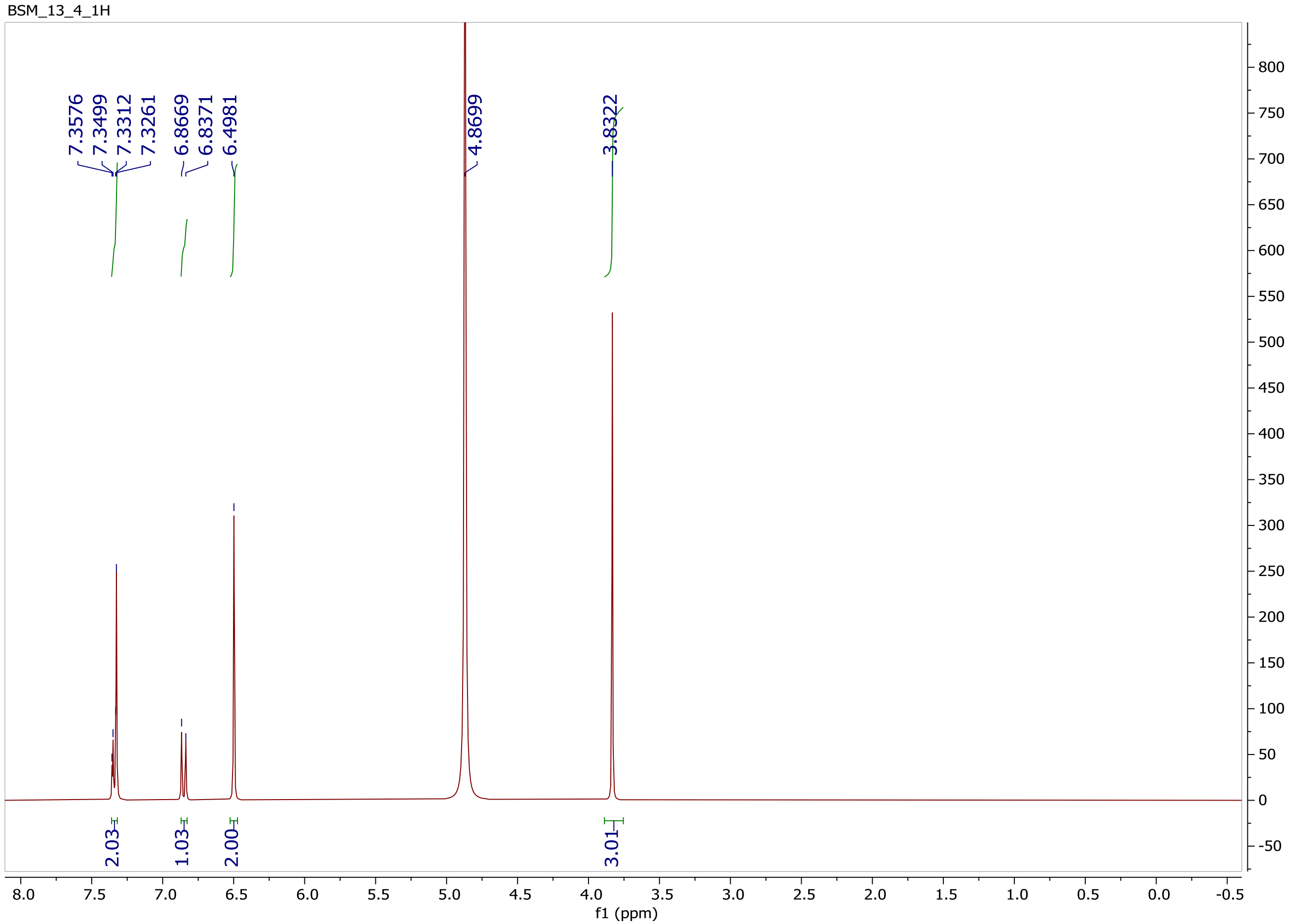

Figure S17. ^{1}H NMR spectrum of compound **5** (δ, 300 MHz, $CDCl_3$).

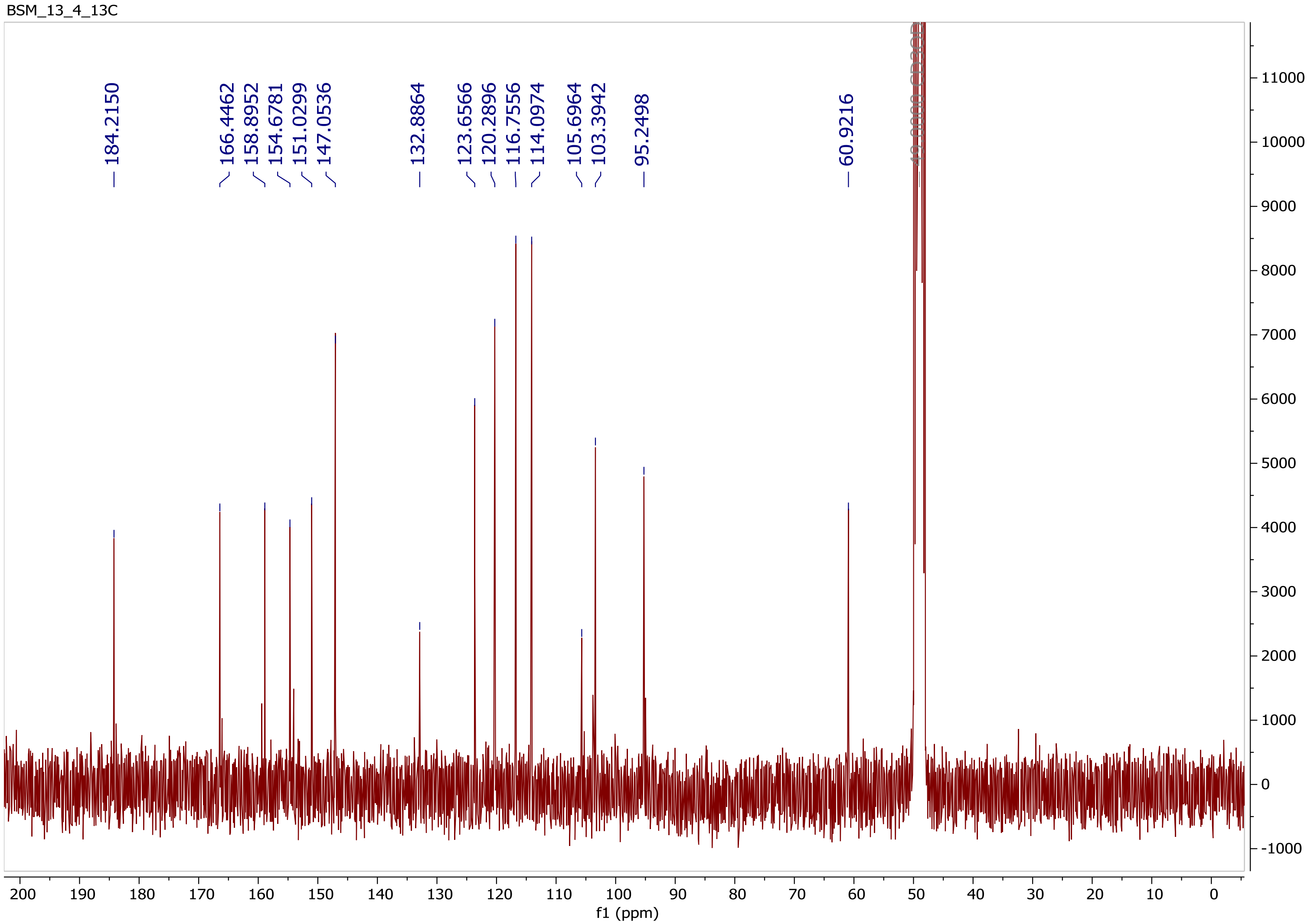

Figure S18. ^{13}C NMR spectrum of compound **5** (δ, 75 MHz, $CDCl_3$)

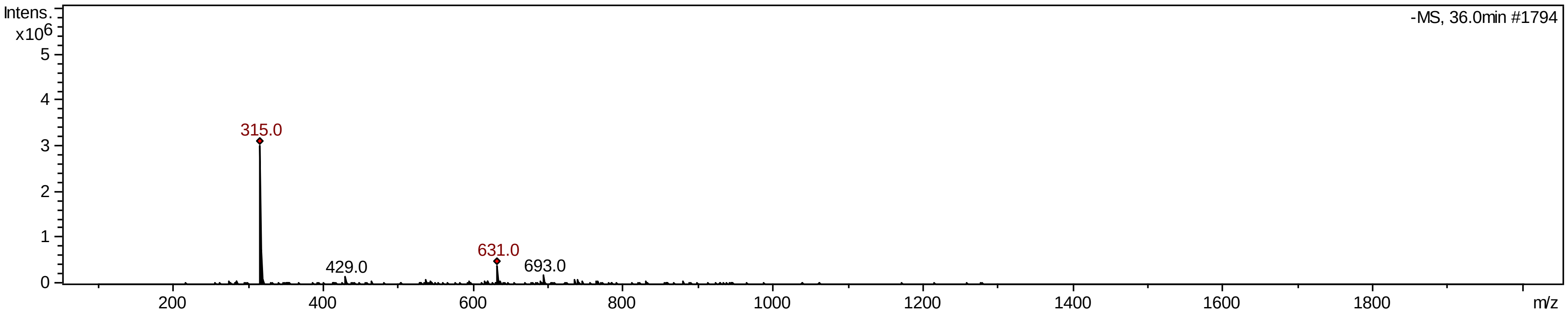

Figure S19. ESI-MS of compound **5** (negative mode).

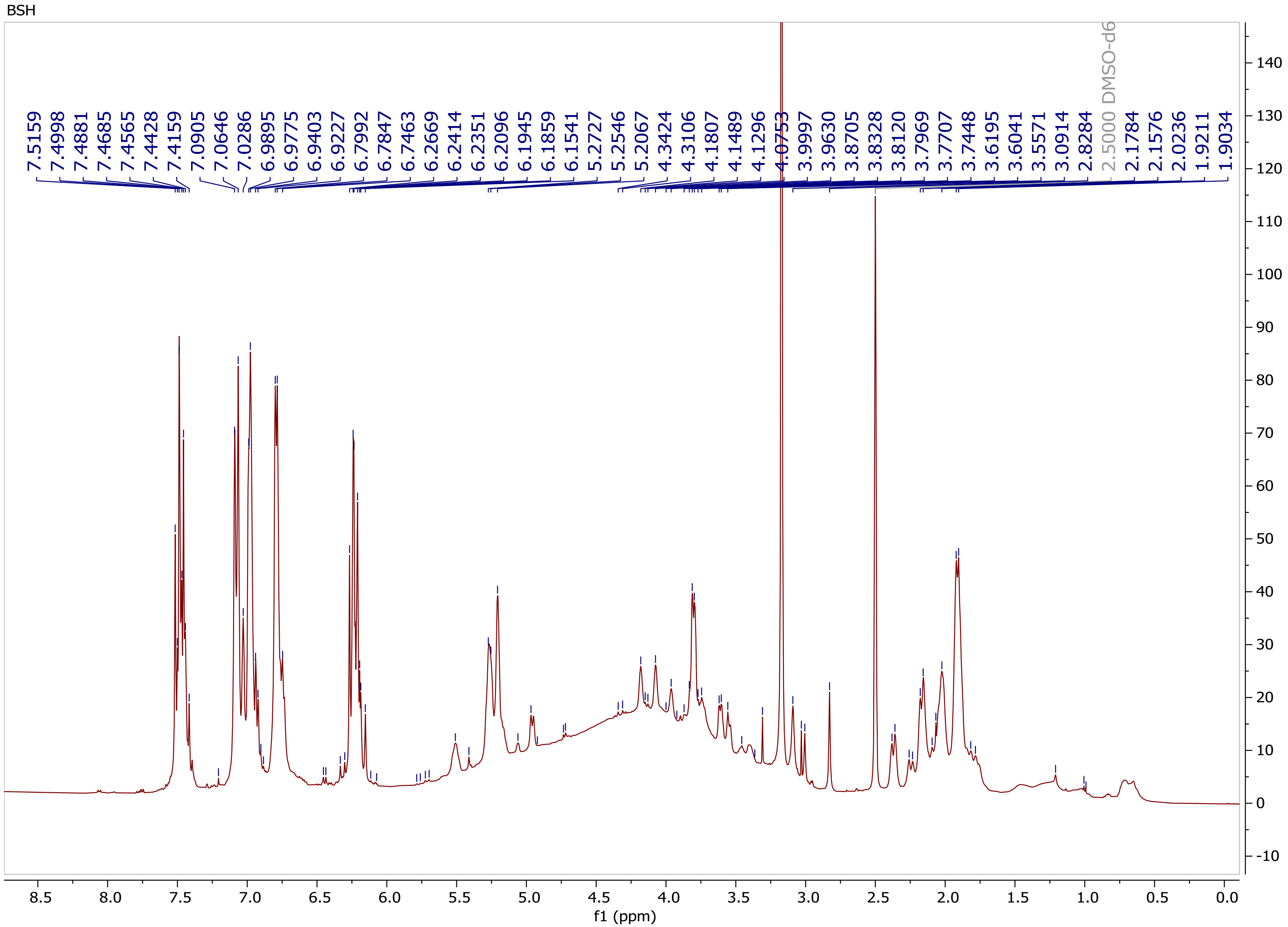

Figure S20. ^{1}H NMR spectrum of compounds **6 – 8** (δ, 300 MHz, DMSO-d_6).

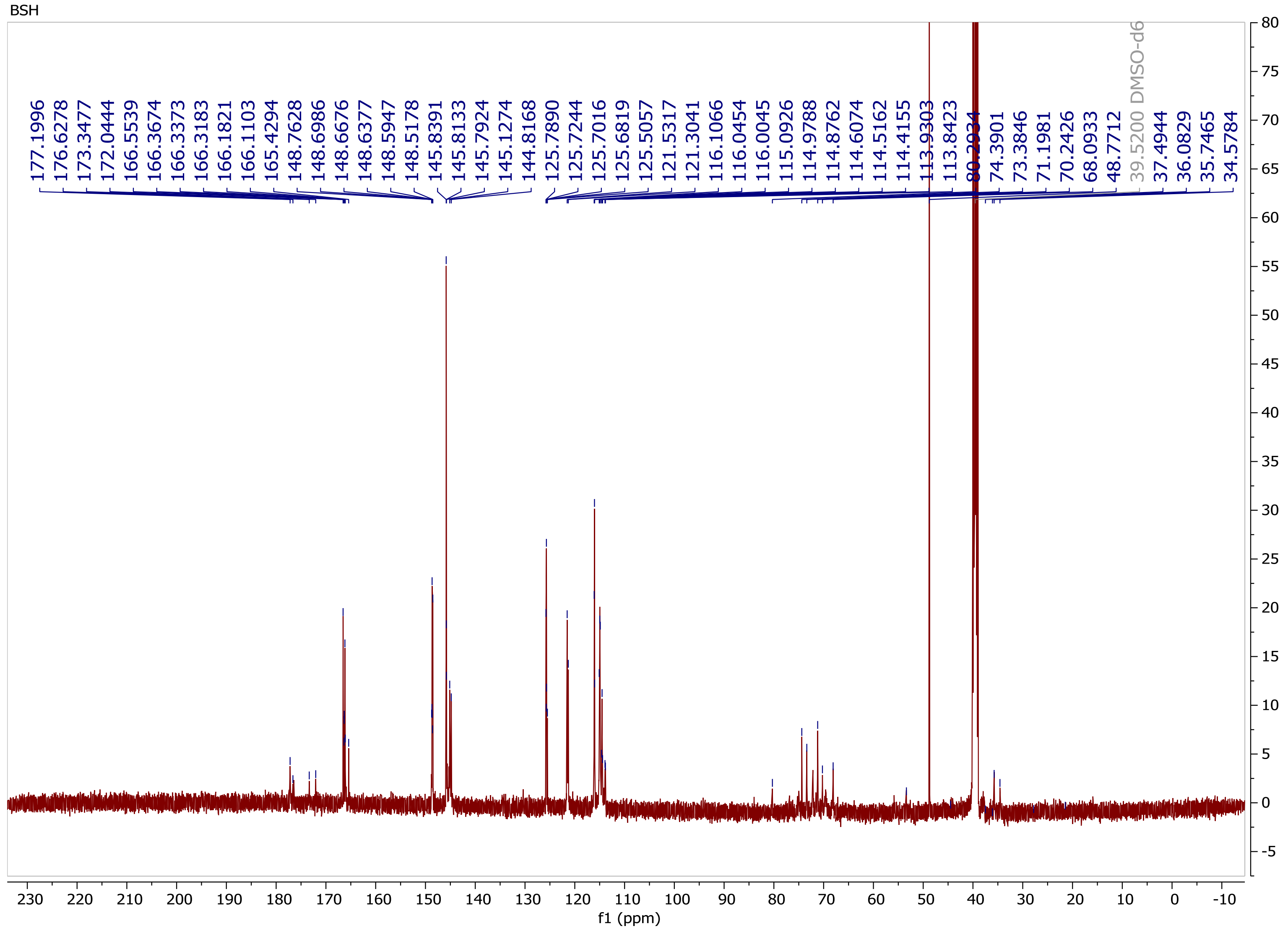

Figure S21. ^{13}C NMR spectrum of compounds **6** – **8** (δ, 75 MHz, DMSO-d_6).

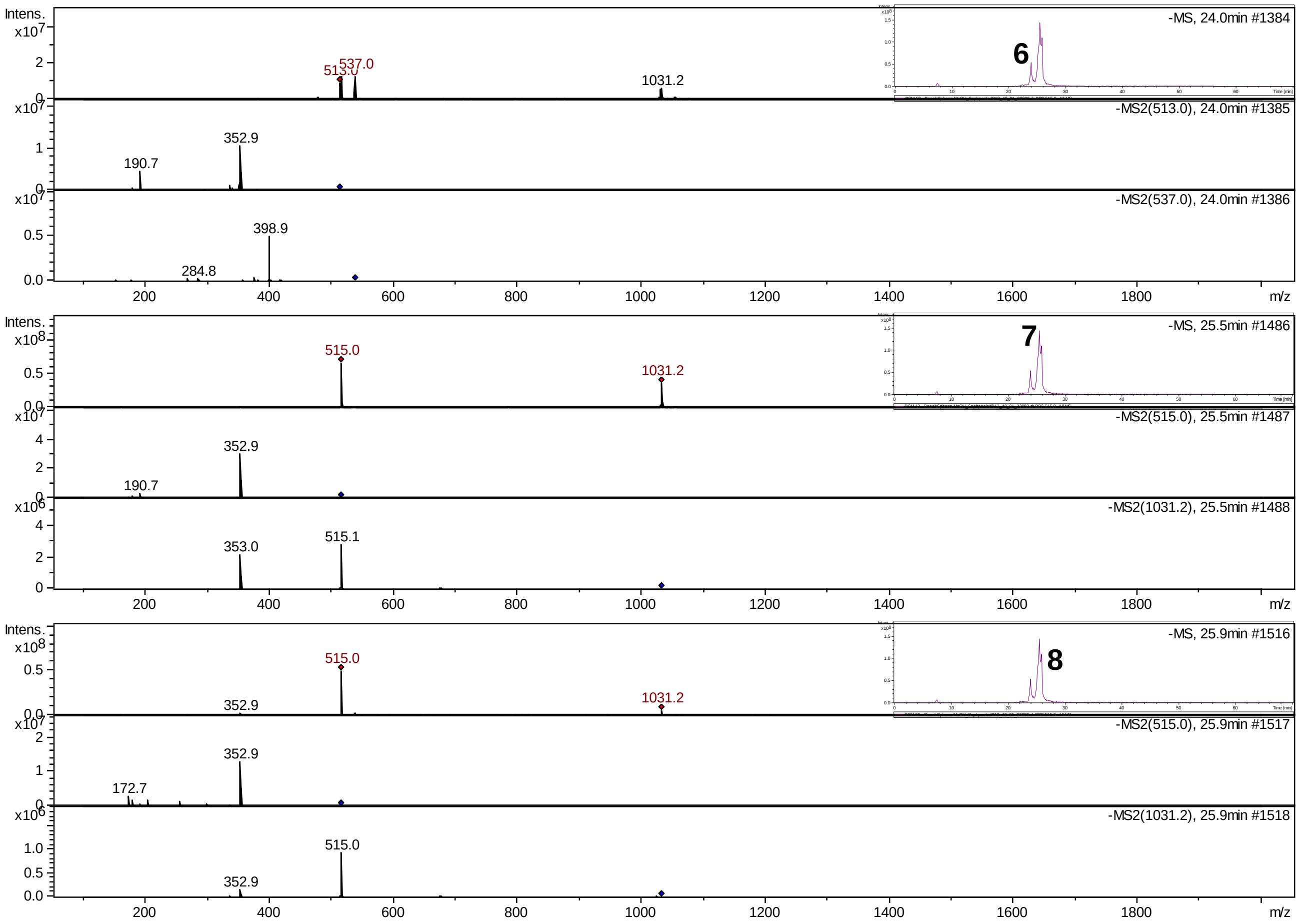

Figure S22. ESI-LRMS spectrum of compounds **6** – **8** (negative mode).
